# Single-molecule studies of high-mobility group B architectural DNA bending proteins

**DOI:** 10.1007/s12551-016-0236-4

**Published:** 2016-11-15

**Authors:** Divakaran Murugesapillai, Micah J. McCauley, L. James Maher, Mark C. Williams

**Affiliations:** 1 Department of Physics, Northeastern University, Boston, MA 02115 USA; 2 Department of Biochemistry and Molecular Biology, Mayo Clinic College of Medicine, Rochester, MN 55905 USA

**Keywords:** HMGB, Binding, DNA, Protein, Bending, Kinetics

## Abstract

Protein–DNA interactions can be characterized and quantified using single molecule methods such as optical tweezers, magnetic tweezers, atomic force microscopy, and fluorescence imaging. In this review, we discuss studies that characterize the binding of high-mobility group B (HMGB) architectural proteins to single DNA molecules. We show how these studies are able to extract quantitative information regarding equilibrium binding as well as non-equilibrium binding kinetics. HMGB proteins play critical but poorly understood roles in cellular function. These roles vary from the maintenance of chromatin structure and facilitation of ribosomal RNA transcription (yeast high-mobility group 1 protein) to regulatory and packaging roles (human mitochondrial transcription factor A). We describe how these HMGB proteins bind, bend, bridge, loop and compact DNA to perform these functions. We also describe how single molecule experiments observe multiple rates for dissociation of HMGB proteins from DNA, while only one rate is observed in bulk experiments. The measured single-molecule kinetics reveals a local, microscopic mechanism by which HMGB proteins alter DNA flexibility, along with a second, much slower macroscopic rate that describes the complete dissociation of the protein from DNA.

## Introduction

The control of gene expression necessary for cells to survive is effected to a great extent by controlling the accessibility of genetic information to RNA polymerase. In mitochondria, organelles that are devoid of histone proteins, the genetic material is preserved in a compact form by mitochondrial transcription factor A (TFAM) and Abf2p in human cells and in yeast, respectively (Bogenhagen et al. 2003, 2008; Friddle et al. 2004; Kang et al. 2007; Kaufman et al. 2007; Lodeiro et al. 2012; Parisi et al. 1993; Rubio-Cosials and Solà 2013; Spelbrink 2010). In eukaryotic cells, nuclear DNA is packaged into chromatin by wrapping onto histone octamers to form nucleosomes. This basal chromatin structure can be modified by various chromatin-associated proteins, altering access to genomic DNA for gene regulation (Albert et al. 2012, 2013; Berger et al. 2007; Hall et al. 2006; Merz et al. 2008; Venema and Tollervey 1999; Wittner et al. 2011). Here, we review the biophysics of one such class of chromatin-associated proteins, the high-mobility group B (HMGB) family, which contain one or two HMGB DNA binding motifs known as boxes. These proteins are known to modify chromatin structure and to bend DNA, as determined by single-molecule studies. The general characteristics of HMGB proteins have also been comprehensively reviewed elsewhere (Malarkey and Churchill 2012).

HMGB proteins are highly abundant eukaryotic nuclear DNA bending proteins, exceeded in abundance only by nuclear histones (Albert et al. 2013; Bianchi 2009; Crothers 1993; Lange et al. 2008; Liu et al. 2010; Sebastian et al. 2009; Štros 2010). Many HMGB proteins are known to bind non-sequence-specifically into the minor groove and to sharply kink DNA (Dragan et al. 2003, 2004; Klass et al. 2003; Thomas and Travers 2001). As for most DNA binding proteins, binding to DNA is typically driven entropically by the release of condensed counterions from the nucleic acid upon electrostatic interaction with the protein. This is supplemented by van der Waals contacts, water release, and both direct and water-mediated hydrogen bonding. Intercalation unwinds and induces a strong, continuous bend in the double helix (Murphy et al. 1999; Thomas and Travers 2001). Despite their abundance, the biological functions of HMGB proteins remain unclear. It is hypothesized that nuclear HMGB proteins facilitate access to genomic DNA by replacing, or changing the structure of, nucleosomes, which are the basic unit of chromatin. The striking ability of HMGB proteins to bind and bend DNA suggests that enhancement of apparent DNA flexibility may also play a biological role (Ragab and Travers 2003; Štros 2010; Travers 2003). It has long been known that HMGB proteins can accelerate the ligase-catalyzed cyclization of DNA fragments into small circles (Paull et al. 1993; Pil et al. 1993; Ross et al. 2001). Because the rate of cyclization of such fragments is limited by DNA flexibility, such cyclization enhancement can be considered evidence that HMGB proteins enhance the *apparent* flexibility of DNA. The effect was historically described as a change in apparent flexibility because cyclization acceleration could arise simply by HMGB promotion of more condensed DNA structures with reduced end-to-end distances even without increasing the actual flexibility of the chain. Hence, the biophysical mechanism by which HMGB proteins alter apparent DNA flexibility has been a subject of significant interest (Bianchi and Agresti 2005; Farge et al. 2012; Gerlitz et al. 2009; McCauley et al. 2007; Skoko et al. 2004; Stefanovsky et al. 2001; Zhang et al. 2009, 2012). Here, we review single-molecule characterizations of HMGB architectural DNA bending proteins, including the recent discovery of both macroscopic and microscopic binding mechanisms that describe HMGB–DNA interactions.

## Single-molecule experiments

**Fig. 1:**
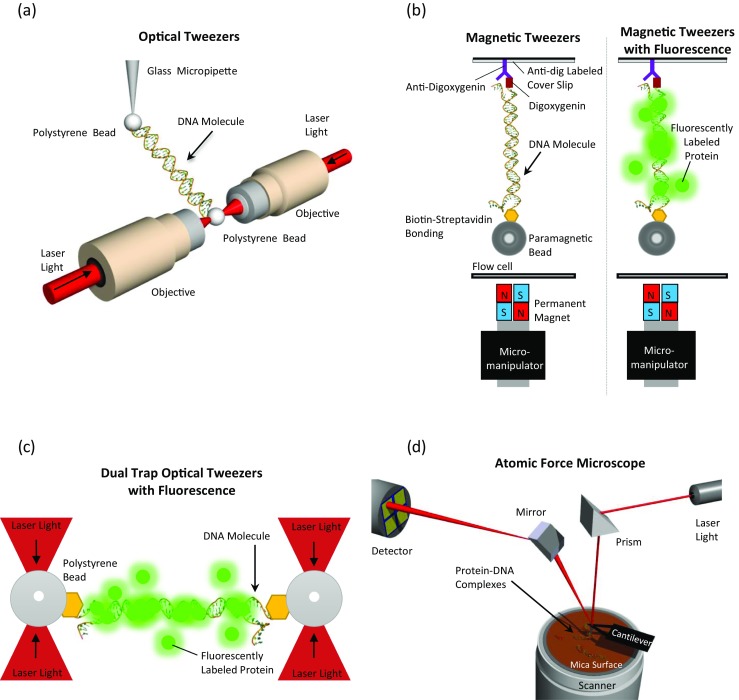
Schematic illustrations (not to scale) depicting single-molecule techniques used to investigate HMGB architectural protein binding to DNA. Optical tweezers, magnetic tweezers and atomic force microscopy are used. **a** In an optical tweezers setup, DNA tethered between labeled beads is extended and released. A glass micropipette tip is used to extend the DNA molecule, while on the other extremity, the deflection of the laser beam during extension is recorded and the signal is then translated into force. (From Murugesapillai et al. 2014). **b** In a magnetic tweezers setup, DNA tethered between a labeled paramagnetic bead and a functionalized cover slip is held at constant magnetic force and the extension is recorded using a CCD camera. Magnetic tweezers combined with fluorescently labeled proteins (*green*) allows visualization as well as quantification of protein binding. (Adapted from Skoko et al. 2004 and Xiao et al. 2010). **c** In a dual trap optical tweezers setup, DNA tethered between labeled polystyrene beads is extended and released. Fluorescently-labeled molecules (*green*) interact with the DNA and their binding can be visualized. (Adapted from Heller et al. 2014). **d** Atomic force microscopy is used to visualize protein–DNA complexes. The reflection of the laser beam off the cantilever to detector is then converted into an imaging signal. (Adapted from Murugesapillai et al. 2014)

**Fig. 2:**
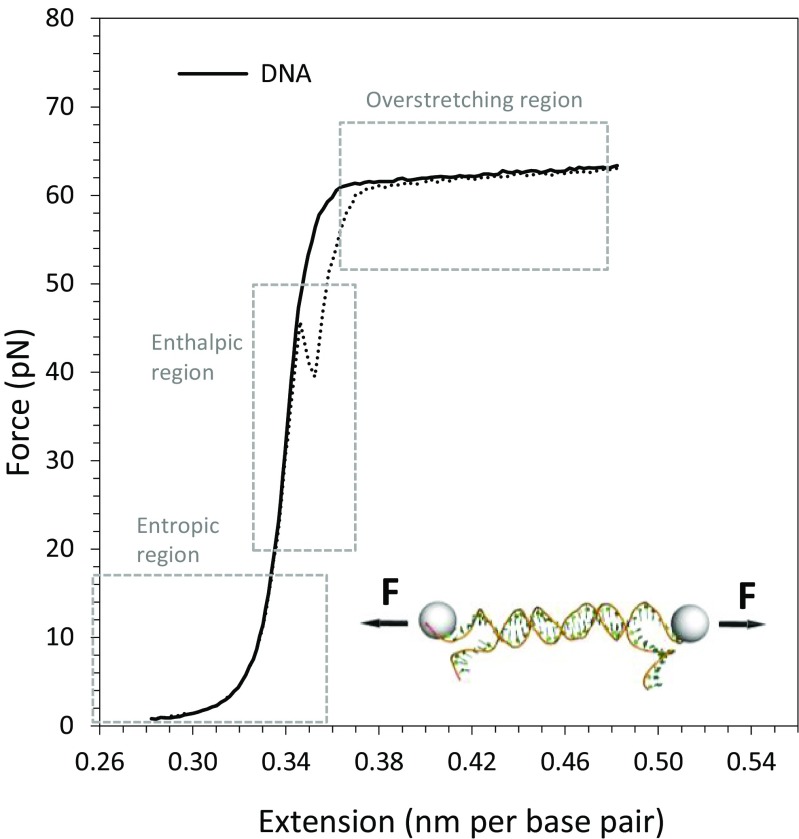
Extension and release of a bacteriophage λ DNA. **a** Measured extension (*solid black*) and release (*dotted black*) curves of bacteriophage λ DNA (48,500 base pairs). (Adapted from McCauley et al. 2013; Murugesapillai et al. 2014)

Optical tweezers (Ashkin et al. 1990; Bustamante et al. 2003; Heller et al. 2014; McCauley and Williams 2009; Neuman and Block 2004) have been used to stretch single DNA molecules in the presence or absence of HMGB proteins (McCauley et al. 2005, 2007, 2013; Murugesapillai et al. 2014). In studies using dual beam optical tweezers, two high-power laser beams are focused onto a small diffraction-limited spot of ∼1 μm. Any object whose index of refraction is greater than that of the surrounding water (*n* = 1.33), will be trapped due to a radiation force that pushes the bead to the center of the resulting trap. A streptavidin-coated polystyrene bead (refractive index *n* = 1.55) is attracted to the focus of the spot. A biotinylated DNA is tethered between this bead and another that is immobilized on a micropipette tip, shown in Fig. 1a (Chaurasiya et al. 2010; McCauley and Williams 2009; Neuman and Block 2004). Single DNA molecules can be thus stretched and characterized, as shown in Fig. 2. In order to characterize the interaction of proteins with such tethered DNA molecules, a solution with a fixed protein concentration is allowed to flow into the experimental cavity surrounding the DNA. Thus, the DNA provides a lattice of binding sites for sequence non-specific DNA binding proteins. Bound proteins alter the DNA stretching curves, allowing binding kinetics and energetics to be characterized using the methods discussed below (Chaurasiya et al. 2010; Heller et al. 2014; McCauley and Williams 2009).

In addition to optical tweezers, magnetic tweezers can also be used to characterize DNA–protein binding, as shown in Fig. 1b. Instead of an optical trap, for which the force is proportional to the distance from the trap, magnetic tweezers use magnetic force to stretch DNA at a constant force (Chen et al. 2011; De Vlaminck and Dekker 2012; Gosse and Croquette 2002; Skoko et al. 2004). While optical tweezers provide a distance clamp with a weak, harmonic trap, magnetic tweezers provide an intrinsic force clamp due to the exponential drop of the force by the magnet on the bead. A single DNA molecule is tethered between a cover slip at one end and a paramagnetic bead on the other end. By moving the permanent magnet, the force acting on the bead can be controlled and recorded by tracking the motion of the bead in the *x*–*y* plane, as shown in Fig. 1b. Furthermore, magnetic tweezers can also be combined with fluorescence to visualize and quantify the binding of proteins to a single molecule of DNA at low forces, as shown in Fig. 1b (De Vlaminck and Dekker 2012; Giuntoli et al. 2015; Graham et al. 2011).

To probe the binding of proteins to a single DNA molecule, dual trap optical tweezers experiments have been combined with detection of fluorescently labeled proteins (Heller et al. 2014), as shown in Fig. 1c. This technique allows characterization of the effects of protein binding on DNA force–extension measurements described above for optical tweezers, while simultaneously determining the distribution of proteins along the DNA molecule as well as the numbers of proteins bound at specific locations. Such measurements can provide additional information about the cooperativity of protein binding as well as the ways in which DNA can be reorganized through protein interactions (Heller et al. 2014). These measurements can be done at single-molecule resolution, including at high concentrations by using stimulated emission depletion microscopy (Heller et al. 2013).

To complement DNA stretching techniques, atomic force microscopy (AFM) imaging is used to directly measure protein-bound sites on a single DNA molecule from the topology of a DNA–protein complex on a surface. These complexes are deposited on a mica surface and scanned, thus allowing the conformation of these complexes to be visualized and quantified. In addition to determining the location and distribution of proteins bound to DNA, AFM provides important information on the nature of the DNA bends induced by proteins.

In the following sections, we will describe how each of these methods can be used to determine both equilibrium and non-equilibrium interactions of HMGB proteins with DNA. Equilibrium measurements allow one to extract equilibrium protein–DNA binding affinities, binding cooperativities, and overall DNA bending characteristics. Non-equilibrium measurements allow the determination of protein association and dissociation rates. In addition, we will show that the dissociation rates can be separated into macroscopic and microscopic components.

## Equilibrium HMGB protein–DNA interactions

### Analysis of DNA force–extension measurements

Experimental data curves for extension and release of a single double-stranded DNA (dsDNA) molecule are displayed in Fig. 2. In the example shown, the DNA is extended in a buffer containing 10 mM Hepes, with pH 7.5 and 100 mM Na^+^. Forces measured in picoNewtons (pN) are plotted as a function of the total extension distance divided by the number of base pairs (nm/bp). Since the distance between two consecutive dsDNA base pairs is 0.34 nm, at an extension of 0.34 nm/bp, the contour length of the dsDNA is reached as the DNA is straightened and becomes taut. The region at forces below 10 pN is termed the entropic regime because DNA can assume many conformations with equal energy, and extending dsDNA decreases the conformational entropy. In this regime, the extension length is shorter than the contour length and the force increase for a given extension increase is small. One parameter used to describe polymer elasticity is the persistence length, *P*, which is related to the distance along the molecule over which angular correlations are lost (Storm and Nelson 2003). Stiffer polymers have longer persistence lengths. Unlike single-stranded DNA (ssDNA), dsDNA is a particularly stiff polymer. The persistence length of dsDNA is ~50 nm, corresponding to ∼150 base pairs (15 turns of the double helix). The persistence length of ssDNA is ∼0.7 nm, two orders of magnitude smaller than for dsDNA, representing just 2 bases, and reflecting the high flexibility of ssDNA (Smith et al. 1996). Once the contour length of 0.34 nm/bp is reached during the stretching of dsDNA, the force at a given extension increases more rapidly, defining the enthalpic regime. In this region, dsDNA displays the elastic characteristics of a polymer, both due to the response of the sugar phosphate backbone and to a major response of the base stacking to the stretching force (Marko and Siggia 1995). The force versus extension curve now follows Hooke’s law, explaining why this region is alternatively termed the elastic regime. Both the elastic and entropic regimes are well described by the high force approximation of the Extensible Worm-Like Chain (WLC) model (Baumann et al. 1997; Marko and Siggia 1995; Odijk 1995; Podgornik et al. 2000; Wenner et al. 2002)

1 $$ {b}_{ds}(F)={B}_{ds}\left[1-\frac{1}{2}{\left(\frac{k_bT}{P_{ds}F}\right)}^{\raisebox{1ex}{$1$}\!\left/ \!\raisebox{-1ex}{$2$}\right.}+\frac{F}{S_{ds}}\right], $$

where *b*_*ds*_ and *F* are the measured extension and force, respectively, *P*_*ds*_ is the persistence length, *B*_*ds*_ is the contour length of the DNA measured in the unit of nm/bp, and *S*_*ds*_ represents the elastic modulus, which takes into account the backbone extensibility. At approximately 65 pN of stretching force, a clear transition is observed, where the length of the DNA has nearly doubled and the force remains essentially constant (Cluzel et al. 1996; Smith et al. 1996; Williams et al. 2002). This plateau region is called the overstretching transition. In this region of force-induced DNA melting, the DNA unwinds and many base pairs between DNA strands are lost broken. Some base pairing in the most stable GC-rich regions is preserved, allowing reversible reannealing as stretching force is reduced. Some hysteresis is observed, as indicated by the dotted curve in Fig. 2. If a DNA molecule is stretched further, to about 1.7 times its contour length, at a force above ∼150 pN in 100 mM Na^+^, the two strands will fully separate, assuming the DNA is tethered to the beads by opposite strands. (McCauley and Williams 2009). The exact form of the DNA during the overstretching transition, whether it reflects force-induced melting or a transition to another double-stranded state, depends strongly on solution conditions and attachment geometry (Bianco et al. 2011; Bongini et al. 2014a, b; Bosaeus et al. 2012, 2014; Fu et al. 2010; King et al. 2013; Paik and Perkins 2011; Shokri et al. 2008; van Mameren et al. 2009; Williams et al. 2001a, b, 2002; Zhang et al. 2013). However, it is clear that dsDNA binding proteins such as HMGB proteins, as well as intercalating small molecules, stabilize the dsDNA structure, resulting in increased overstretching force as more ligands are bound to the dsDNA molecule (Almaqwashi et al. 2016; Chaurasiya et al. 2010; McCauley et al. 2005, 2007, 2008, 2013). Thus, dsDNA binding by proteins or other ligands must be disrupted during overstretching.

### Single box and double box HMGB proteins alter the mechanical properties of DNA

**Fig. 3:**
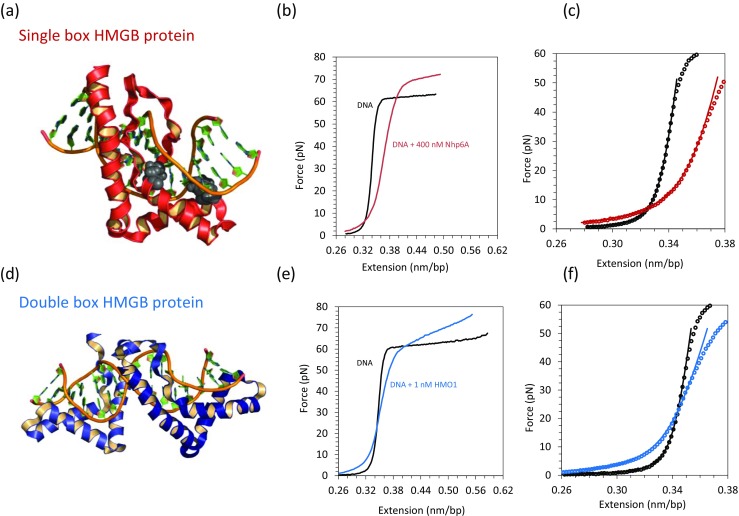
Binding of Nhp6A and HMO1 proteins to λ DNA characterized by optical tweezers. **a** Solution structure of the yeast single box Nhp6A protein bound to DNA with intercalating amino acid side chains shown as *gray* space-filled atoms (PDB code: 1J5N). **b** Force–extension curves are shown for phage λ DNA in the absence (*black*) and presence (*red*) of the single box Nhp6A protein. **c** Fits to the WLC model in the absence (*black*) and presence (*red*) of Nhp6A. **d** Solution structure of a double box HMGB protein bound to DNA (PDB code: 2GZK). **e** Force–extension curves are shown for phage λ DNA in the absence (*black*) and presence (*blue*) of the double box HMO1 protein. **f** Fits to the WLC model in the absence (*black*) and presence (*blue*) of HMO1. (Adapted from McCauley et al. 2013; Murugesapillai et al. 2014)

For comparison of single and double box HMGB proteins, we will first discuss the single box HMGB protein yeast Nhp6A and the double box HMGB protein yeast HMO1 (Allain et al. 1999; McCauley et al. 2005, 2007, 2013; Murugesapillai et al. 2014; Paull et al. 1996; Skoko et al. 2004). Figure 3a shows the solution NMR structure of the Nhp6A protein (PDB code: 1J5N). The three alpha helices are somewhat disordered before binding to DNA. A strong bend is induced in the DNA upon protein binding into the minor groove with partial intercalation, altering base pair stacking and leading to partial DNA unwinding.

In studies of Nhp6A, a 400 nM solution of Nhp6A protein was introduced into the buffer solution surrounding bacteriophage λ DNA tethered in an optical tweezer apparatus. The protein–DNA complexes were allowed to chemically equilibrate. The subsequent stretching and release data collected in the presence of Nhp6A are shown in red in Fig. 3b along with the protein-free DNA data (in black) to facilitate comparison (McCauley et al. 2013).

In the presence of HMGB proteins such as Nhp6A, the force–extension curve (in red) is above the DNA-only curve (in black) in the entropic region. This is due to protein-induced DNA compaction as well as a reduction in the DNA persistence length, resulting in DNA–protein complexes that are shorter than free DNA at low forces. At stretching forces above 10 pN, the contour length of Nhp6A-saturated DNA is actually longer than DNA alone, presumably due to intercalation, as illustrated in Fig. 3b, c. This observation is consistent with the solution NMR structure showing intercalation, shown in Fig. 3a. The overstretching transition force increases up to 73 pN, interpreted as Nhp6A stabilization of dsDNA, due to preferential binding to dsDNA relative to ssDNA, as shown in Fig. 3b. The extension and release curves are very similar, suggesting that the protein does not fully dissociate during stretch and release (time scale longer than 100 s). Even after applying a force up to 200 pN, HMGB proteins were not observed to dissociate, in contrast to what would be expected for pure DNA bending proteins, which shorten DNA in a process that is inhibited by force (McCauley et al. 2013). The observed DNA behavior in the presence of HMGB proteins is consistent with the fact that these proteins also intercalate, elongating the DNA in a process that is favored by force (Farge et al. 2012; McCauley et al. 2005, 2007; Zhang et al. 2009, 2012)

Figure 3d shows the solution NMR structure of a double box HMGB protein bound to DNA (PDB code: 2GZK). HMO1, another double box HMGB protein (Albert et al. 2013; Bauerle et al. 2006; Kamau et al. 2004), induces a force–extension curve that is above the DNA-only curve below 20 pN of stretching force (Murugesapillai et al. 2014), as illustrated in Fig. 3e, f. The double box HMGB mitochondrial regulatory protein TFAM displays similar effects (Farge et al. 2012). These effects illustrate the compacting, bending and force-facilitated intercalating nature of these proteins. Similar to single box Nhp6A, the double box HMO1 stabilizes double-stranded DNA, which is illustrated by the increase of the overstretching transition force, as shown in Fig. 3e.

**Fig. 4:**
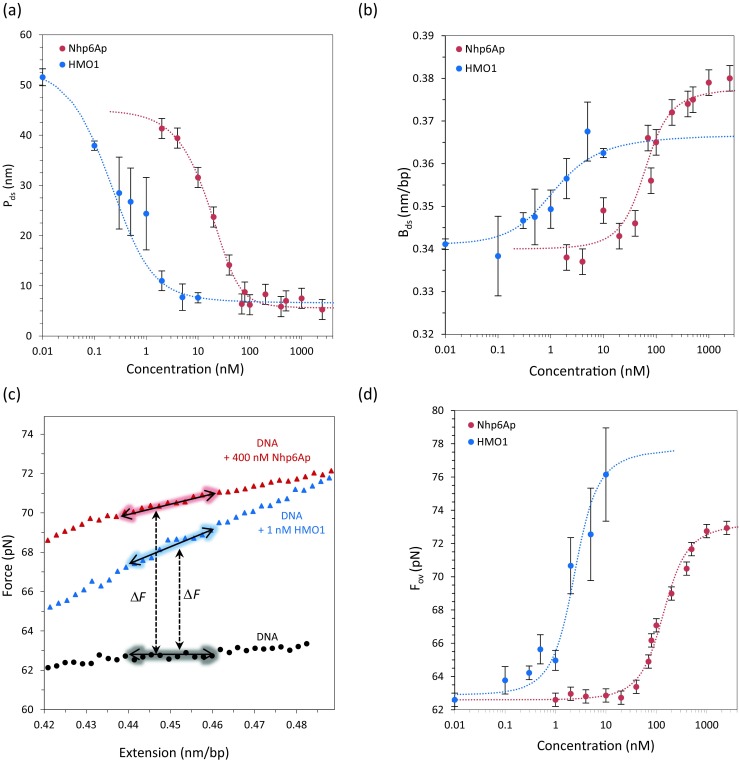
Equilibrium analysis of Nhp6A and HMO1 protein binding to DNA. **a** Persistence length of the DNA in the presence of Nhp6A (*red*) and HMO1 (*blue*) as a function of concentration is fitted to Eqs. 2 and 4 to obtain *K*_D_ = 71 ± 14 nM and *ω* = 20 for Nhp6A, and *K*_D_ = 2.1 ± 0.8 nM and *ω* = 20 ± 7 for HMO1. **b** Contour length of DNA in the presence of Nhp6A (*red*) and HMO1 (*blue*) as a function of concentration is fitted to Eqs. 2 and 6 to obtain *K*_D_ = 71 ± 14 nM and *ω* = 20 for Nhp6A, and *K*_D_ = 1.9 ± 0.7 nM and *ω* = 18 ± 5 for HMO1. **c** The DNA overstretching region with extensions only is shown for DNA in the absence (*black circles*) and presence of Nhp6A (*red triangles*) and HMO1 (*blue triangle*). (Adapted from McCauley et al. 2013; Murugesapillai et al. 2014). **d** Overstretching force is fitted to the site exclusion binding isotherm of Eqs. 2 and 3, yielding measurements of *K*_D_ = 160 ± 20 nM and *ω* = 20 for Nhp6A, and *K*_D_ = 2.8 ± 0.6 nM and *ω* = 80 ± 15 for HMO1

These data can be fit to the WLC model given in Eq. (1) and the elastic properties of the DNA–protein complexes can be extracted. Saturation (the protein concentration above which the persistence length does not change) is reached at 400 nM for Nhp6A, 550 nM for HMGB2, 50 nM for TFAM, and 10 nM for HMO1 proteins (Farge et al. 2012; McCauley et al. 2013; Murugesapillai et al. 2014). Interestingly, these results show that double box HMGB proteins have higher affinity for DNA compared to single box proteins. To gain more insight into the mechanical properties of the HMGB–DNA complexes, the elastic response of the dsDNA polymer in the absence and in the presence of HMGB proteins is quantitated by fitting to the WLC model. The upper limit used for the fit is ∼30 pN, chosen to avoid twist–stretch coupling due to DNA unwinding (Gross et al. 2011). Figure 3c represents fits to the WLC model in the absence (black) and presence (red) of 400 nM Nhp6A. Figure 3f represents fits to the WLC model in the absence (black) and presence (blue) of 1 nM HMO1. The persistence length obtained by fitting the data in the presence of saturating concentrations of Nhp6A proteins is 5.5 ± 0.5 nm, remarkably reduced from the ∼50 nm of DNA only (Table 1). Thus, DNA flexibility in the presence of Nhp6A is drastically altered, on the scale of tens of nm, as seen for ssDNA. This trend remains true for double box HMGB proteins, revealing a powerful function of such proteins in promoting nucleoprotein assemblies. At saturating concentrations, the single box Nhp6A (in red) and the double box HMO1 (in blue) decrease the persistence length of the DNA by 87 and 85 %, respectively, as shown in Fig. 4a. It is interesting to note that, to decrease the persistence length of the DNA by a factor of two, the concentration of double box versus single box differs by one order of magnitude. When the DNA is exposed to HMGB proteins, the effective DNA contour length increases up to 5 % for HMO1, and 12 % for Nhp6A, presumably reflecting the intercalating character of these proteins, as shown in Fig. 4b. Interestingly, as for the persistence length, to increase the effective contour length of the DNA to half of the total amount increased, the concentration of the double box and single box differs by more than one order of magnitude.

**Table 1:** Comparison of the fit parameters persistence length *P*_*ds*_, contour length *B*_*ds*_, and elastic stretch modulus *S*_*ds*_ of the WLC model, all obtained at saturated protein concentration, as well as the dissociation constant *K*_D_ and the cooperativity parameter ω for single box and double box HMGB proteins

	DNA^a^	DNA + Nhp6A^a^	DNA + HMGB2^a^	DNA + TFAM	DNA + HMO1^d^	DNA + Abf2p
*P*_*ds*_ (nm)	48 ± 2	5.5 ± 0.5	4.6 ± 0.5	4 ± 2^b^	7.6 ± 1.0	
*B*_*ds*_ (nm/bp)	0.340 ± 0.002	0.378 ± 0.002	0.378 ± 0.001	0.367 ± 0.018^b^	0.363 ± 0.001	
*S*_*ds*_ (pN)	1400 ± 100	1000 ± 200	1600 ± 200	1300 ± 500^b^	1360 ± 400	
*K*_D_ (nM)		71 ± 14	180 ± 30	∼4^c^ ∼9^b^	2.3 ± 0.4	∼400^e^ ∼40^f^ ∼1400^g^
*ω*		20	20	70 ± 14^b^	23 ± 4	

^a^(McCauley et al. 2013)

^b^(Farge et al. 2012)

^c^(Kaufman et al. 2007)

^d^(Murugesapillai et al. 2014)

^e^(Cho et al. 2001)

^f^(Brewer et al. 2003)

^g^(Friddle et al. 2004)

Furthermore, for both single box and double box HMGB proteins, the overstretching force increases as the concentration is increased. Figure 4c shows the overstretching force for Nhp6A (in red), HMO1 (in blue) and DNA (in black) for reference. The colored arrows indicate the range over which the average has been done. Δ*F* represents the difference in overstretching forces upon HMGB protein binding. Interestingly, HMO1 stabilizes dsDNA at much lower concentrations, reflecting its higher binding affinity, as shown in Fig. 4d. To quantify these effects on DNA biophysical properties, a DNA lattice binding model is applied, as discussed below (McCauley et al. 2013; McCauley and Williams 2011; Murugesapillai et al. 2014).

### Quantifying HMGB-DNA binding using the McGhee-von Hippel binding isotherm

In the cooperative McGhee–von Hippel binding isotherm, DNA is considered to be a lattice of binding sites where proteins can occlude the occupied binding sites. In this model, the proteins first bind to DNA with an intrinsic equilibrium association constant, *K*_A_, occupying *n* base pairs of the DNA upon binding. The variable *n* is called the occluded binding site size. Once the protein is already bound on the lattice, for another protein to bind next to it, the affinity is enhanced by a factor *ω*, where *ω* is defined as the cooperativity parameter. The cooperative McGhee–von Hippel binding isotherm is given by McGhee (1976), McGhee and von Hippel (1974), and Vologodskii (2015):

2 $$ \varTheta ={K}_Acn\left(\mathsf{1}-\varTheta \right){\left[\frac{\left(\mathsf{2}\omega -\mathsf{1}\right)\left(\mathsf{1}-\varTheta \right)+\varTheta /n-R}{\mathsf{2}\left(\omega -\mathsf{1}\right)\left(\mathsf{1}-\varTheta \right)}\right]}^{n-\mathsf{1}}{\left[\frac{\mathsf{1}-\left(n+\mathsf{1}\right)\cdot \varTheta /n+R}{\mathsf{2}\left(\mathsf{1}-\varTheta \right)}\right]}^{\mathsf{2}} $$

with

$$ R=\sqrt{{\left(1-\left(n+1\right)\cdot \varTheta /n\right)}^2+\frac{4\omega \varTheta }{n}\left(1-\varTheta \right)}. $$

Here, Θ is the DNA fractional site occupancy and *n* is the binding site size. The cooperative equilibrium dissociation constant for the protein binding to the lattice is ${K}_D=1/{K}_A\omega$.

To describe the binding of HMGB proteins to DNA, Eq. 2 is applied. Previous studies can be used to estimate the occluded binding site size based on structural information or biochemical assays. For example, *n* ∼ 7 for a single box HMGB protein, as estimated from crystal structures (Churchill et al. 1999; McCauley et al. 2013) and *n* ∼ 30 for double box proteins HMO1 (Kamau et al. 2004), *n ∼* 30 for TFAM (Farge et al. 2012) and Abf2p (Diffley and Stillman 1992), where all the double box binding site sizes were estimated from footprinting experiments. As an example, this model is applied to the measurements of Fig. 4. To do this, the assumption that the overstretching force is proportional to the fraction of proteins bound is considered, given by

3 $$ {F}_{ov}\left(\varTheta \right)={F}_{ov}^D+\varTheta \cdot \left({F}_{ov}^L-{F}_{ov}^D\right), $$

where *F*_*ov*_^*D*^ is the protein-free value of *F*_*ov*_ and *F*_*ov*_^*L*^ is the protein-saturated value of *F*_*ov*_.

Figure 4c shows that the overstretching force increases in the presence of HMGB proteins. This overstretching transition force measured as a function of protein concentration gives a titration curve that can be fit to Eqs. (2) and (3), assuming a lattice binding model, to yield *K*_*D*_, *ω*, and the saturated overstretching force, as shown in Fig. 4d (Kowalczykowski et al. 1986; McGhee 1976; McGhee and von Hippel 1974; Rouzina and Bloomfield 1998; Schellman 1974).

Assuming that the DNA and protein-bound sites can each be treated as independent flexible hinges, the persistence length can be written as (McCauley et al. 2013; Rouzina and Bloomfield 1998)

4 $$ {P}_{ds}\left(\varTheta \right)=\frac{P_L\cdot {P}_D}{P_L+\varTheta \cdot \left({P}_D-{P}_L\right)}, $$

where *P*_*D*_ is the protein-free value of *P*_*ds*_ and *P*_*L*_ is the protein-saturated value of *P*_*ds*_. This fit also yields *K*_*D*_ and *ω*, as well as *P*_*L*_. Another approach to fit these hybrid DNA–protein complex curves is to assume that the protein complex force–extension curves are a linear combination of a DNA-only curve and a DNA curve when saturated with protein. This allows direct calculation of $\varTheta (c)$ from each force–extension curve (Farge et al. 2012; McCauley et al. 2013) using the following relation.

5 $$ b=\varTheta {b}_L+\left(1-\varTheta \right){b}_D $$

where ${b}_D$ is the protein-free extension, ${b}_L$ is the protein-saturated extension, and *b* is the concentration-dependent measured extension, all as a function of force. The resulting $\varTheta (c)$ curve can then be fit to any binding model. However, the latter method requires a reliable measurement of the force–extension curve for the fully saturated DNA–protein complex. This procedure was used to determine the DNA binding affinity of TFAM, assuming a WLC model for both DNA-only and protein-saturated DNA (Farge et al. 2012). The results obtained from the procedure in Eq. (4) agreed reasonably well with those from Eq. (5), even when fitting Eq. (5) to a linear combination of the WLC (for DNA) and FJC (for protein-coated DNA)(McCauley et al. 2013). Therefore, the results from concentration-dependent fits to force–extension curves do not appear to depend strongly on which of the above methods is used.

Similarly, the contour length is given by

6 $$ {B}_{ds}\left(\varTheta \right)={B}_D+\varTheta \cdot \left({B}_L-{B}_D\right), $$

where *B*_*D*_ is the protein-free value of *B*_*ds*_, and *B*_*L*_ is the protein-saturated value of *B*_*ds*_.

Thus, *K*_*D*_ and *ω* can also be obtained independently by fitting Eqs. (2), (4), (5), or (6) to the concentration-dependence of the force–extension curve in Eq. (1), illustrated in Fig. 4a, b, and d (McCauley et al. 2013; Murugesapillai et al. 2014). The *K*_*D*_ obtained from the different methods are all in reasonable agreement. Interestingly, the cooperativity parameter *ω* allows one to calculate the free energy of protein–protein interactions, given by k_B_Tln(*ω*). Thus, single box and double box HMGB proteins interact with themselves with similar affinity, although their *K*_*D*_ for DNA binding differs by one order of magnitude. The results of fits to this model are shown when available in Table 1. Fits to other, simpler models have also been used to determine binding affinities from force–extension data (Biebricher et al. 2015; Cruceanu et al. 2006).

## AFM studies of DNA interactions with HMGB proteins

### Global flexibility

**Fig. 5:**
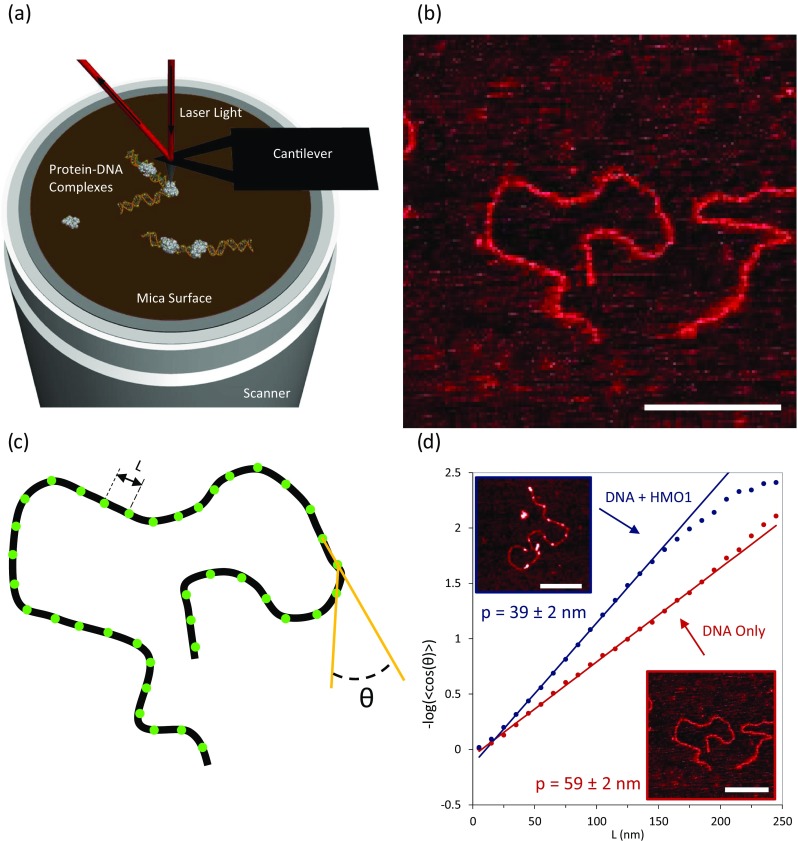
Global flexibility. Binding of double box HMO1 to pBR322 DNA characterized by atomic force microscopy (AFM). **a** Schematic of the AFM instrument used to image DNA–protein interactions. **b** A two-dimensional image illustrates linearized pBR322 DNA on a mica surface (*scale bar* 300 nm). **c** Schematic diagram showing local DNA bend. The angle is calculated from two adjacent line segments (*gold*) drawn between three agacent points, separated by a distance L (*green* dots). **d** A fit to the two-dimensional WLC model (Eq. 7) enables the calculation of DNA persistence length. *Red* and *blue* *curves* correspond to 0.11 nM DNA in the absence (*lower right*; *scale bar* 300 nm) or presence (*upper left* *inset*, *white dots* are bound protein; *scale bar* 200 nm) of 3 nM HMO1 protein. (Adapted from Murugesapillai et al. 2014)

Although optical tweezers allow one to determine the overall average flexibility of a single DNA molecule in the absence and presence of binding proteins, this does not reveal how individual proteins induce changes in flexibility. To determine the effects of local protein binding, atomic force microscopy (AFM) experiments can be used for direct imaging of local DNA bending angles on a surface. A schematic diagram of the experiment is shown in Fig. 5a. HMGB–DNA complexes were imaged in air on a mica surface that had been modified with Mg^2+^ ions as shown in Fig. 5a. The topography of the mica surface decorated with pBR322 DNA only is first obtained, as shown in Fig. 5b. Furthermore, to investigate the effect of HMGB proteins upon binding DNA, HMO1–DNA complexes are imaged, as shown in upper left inset of the Fig. 5d. As described above, global DNA flexibility is defined by the persistence length. To determine the persistence length, *p*, the orientation differences θ along the DNA as a function of contour length segment *L*, as shown in Fig. 5c, are fit to the two-dimensional WLC model (Rivetti et al. 1996; Wiggins et al. 2006).

7 $$ \left\langle \cos \left(\theta \right)\right\rangle ={e}^{-L/2p} $$

In cases where the bend angle orientations are difficult to reliably define, simulations of the DNA bending can also be helpful (Dame et al. 2005). Interestingly, these measurements show that the DNA flexibility increases in the presence of HMO1, with *p* = 39 ± 2 nm (in blue), compared to DNA in the absence of proteins on this surface, where *p* = 59 ± 2 nm (in red), obtained by fitting to Eq. (7), shown in Fig. 5d.

### Local flexibility

**Fig. 6:**
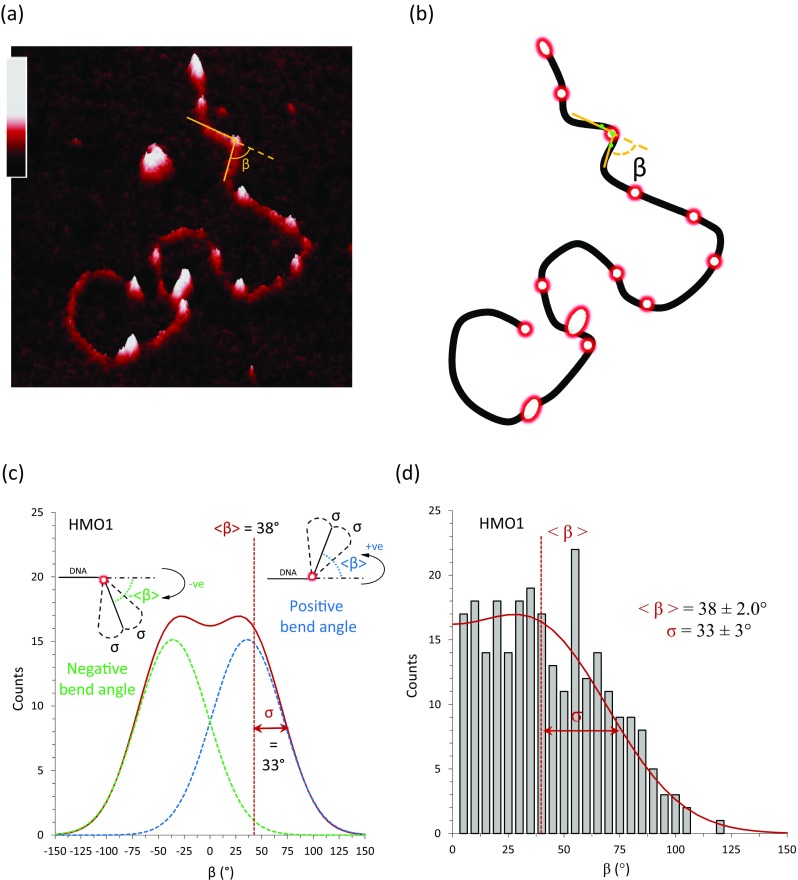
Binding of the double box HMO1 to pBR322 DNA characterized by AFM, illustrating the analysis of local DNA flexibility. **a** A three-dimensional AFM image of HMO1 protein bound to linearized plasmid pBR322 DNA (4361 bp). The *vertical color gradient* *bar* represents the sample height ranging from 0.0 to 2.0 nm. **b** Schematic diagram showing protein-bound locations from DNA only. The angle is calculated from two adjacent line segments (in *gold*) drawn at the location of the protein-bound site (*green dots* are the three equidistant points used to draw the line segments). **c** The measured angle could be either clockwise (positive) or counterclockwise (negative). Both directions are taken into account resulting in a bi-Gaussian fit (*red*), where β is the mean bend angle and σ gives the width of the distribution. **d** Histogram of measured local protein-induced DNA bend angles for the double box HMO1 and fit. The average measured angle is 38 ± 2.0° with σ = 33 ± 3° (Murugesapillai et al. 2014)

Since AFM allows one to resolve protein-bound sites from DNA only, it is now possible to investigate how HMGB proteins increase the apparent flexibility of DNA as well as the nature of the induced bends. A three-dimensional topography of the surface in the presence of HMO1 proteins bound to DNA is shown in Fig. 6a. Protein-bound sites are represented by white peaks along the DNA.

**Fig. 7:**
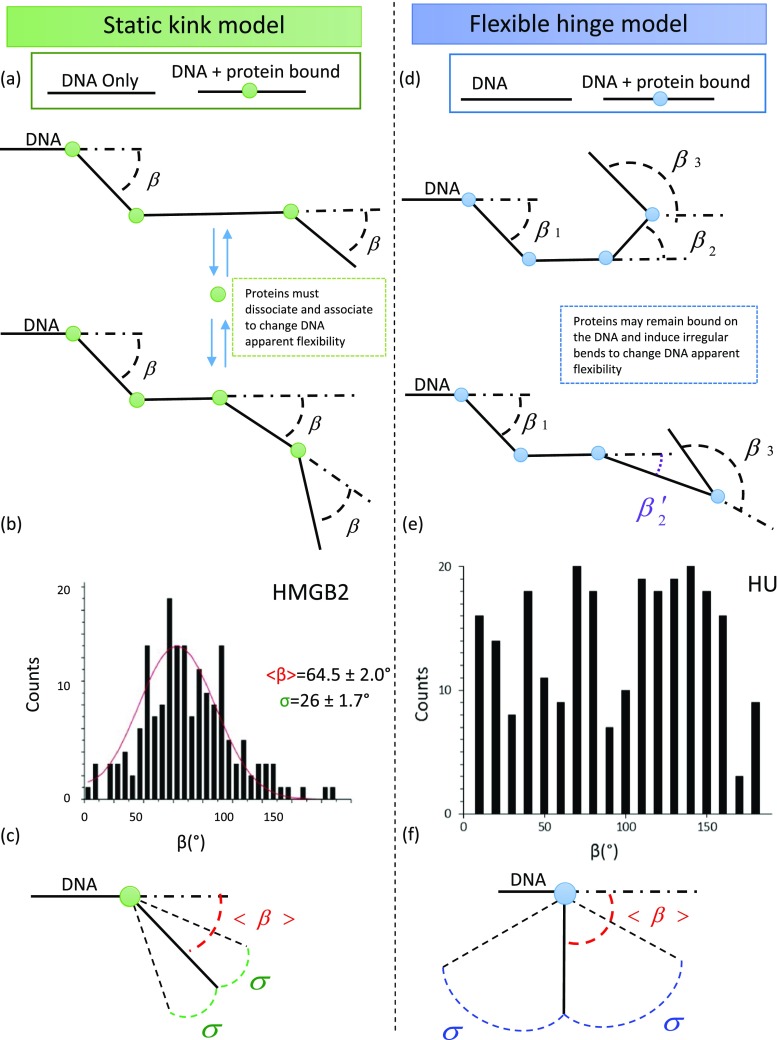
Models describing the nature of local flexibility induced by HMGB proteins upon binding DNA. **a** In the static kink model, the protein binds to DNA and induces a bend angle, *β*. While the protein remains electrostatically bound in the vicinity of the DNA, it can dissociate and associate and each binding event induces the same bend angle, *β*. **b** Measured local protein-induced DNA bend angles for the single box protein human HMGB2 (Box A) and fit (*red*). The average measured angle peaks at 64.5 ± 2.0° with σ = 26.0 ± 1.7°. **c** Model describing the average bend angle and the standard deviation. The narrow standard deviation is indicative of a static kink model. **d** In the flexible hinge model, the protein induces a different bend angle at each binding event, and *β*_2_′ (*purple*) represents a binding event after some time. **e** Measured local protein-induced DNA bend angles for HU proteins. The distribution of angles is very broad. **f** Model describing the average bend angle and the standard deviation. The broad standard deviation is indicative of a flexible hinge model. (Adapted from Zhang et al. 2012 and van Noort et al. 2004)

A protein-induced DNA bending angle, β, is measured at each bound protein site. The green dots represent the equidistant segment length of 50 nm used to draw the two adjacent line segments (in gold), as shown in Fig. 6b. The measured angle could be either clockwise (positive) or counterclockwise (negative). Both directions are taken into account resulting in a bi-Gaussian fit (in red), as shown in Fig. 6c (Murugesapillai et al. 2014; Zhang et al. 2012). The measure of protein-induced DNA bending angle resulted in a histogram with a moderately broad distribution (Fig. 6d). This is significantly different from the results observed for one study of HU proteins, which reported a flat distribution of angles, shown in Fig. 7e (van Noort et al. 2004).

By fitting the bend angle distribution to a bi-Gaussian function, the average bend angle β and the standard deviation σ can be determined. The standard deviation σ illustrates the extent to which the DNA is flexible around the average angle β. A smaller value of σ means the bends are more likely to be near the average bend angle and a larger σ means that the bends are distributed more widely around the average bend angle. The standard deviation of the distribution, σ, was determined to be 33 ± 3° and β averaged 38 ± 2.0° for the double box HMO1, as shown in Fig. 6d (Murugesapillai et al. 2014). Interestingly, AFM studies carried out on a dried surface revealed bending angles of 100 ± 20° for TFAM and 78° for Abf2p (Friddle et al. 2004; Kaufman et al. 2007; Parisi et al. 1993). For comparison, in the absence of protein, the standard deviation of DNA bending angles is about 24° centered at zero degrees (Rivetti and Codeluppi 2001; Zhang et al. 2009, 2012).

## Non-equilibrium binding and kinetics measurements

### Static kink and flexible hinge models

Force–extension measurements and AFM imaging allow characterization of the increased flexibility of DNA in the presence of HMGB proteins. It is now interesting to compare specific models to determine the biophysical mechanism by which HMGB proteins accomplish this important task. In particular, the data distinguishing the two prevailing models for this effect, referred to as the “static kink” and “flexible hinge” models (McCauley et al. 2005; van Noort et al. 2004), are reviewed.

In the static kink model, the protein binds to DNA and induces a bend angle, β. While the protein remains electrostatically bound in the vicinity of the DNA, it experiences cycles of dissociation and re-association such that each binding event induces the same bend angle β at a new position. By random introduction of these static kinks upon binding DNA, these proteins endow the DNA with greater *apparent* flexibility over many binding–unbinding cycles, as shown in Fig. 7a. Thus, any two DNA sites experience higher local concentration. A histogram of measured local protein-induced DNA bend angles for the single box protein human HMGB2 and fit (in red) is shown in Fig. 7b. The average measured angle peaks at 64.5 ± 2.0° with σ = 26.0 ± 1.7°. Thus, for the single box HMGB2, the range of DNA bend angles around the protein-induced DNA bend is not greater than that expected for DNA alone. This narrow standard deviation illustrates the static kink model, as shown in Fig. 7c.

**Fig. 8:**
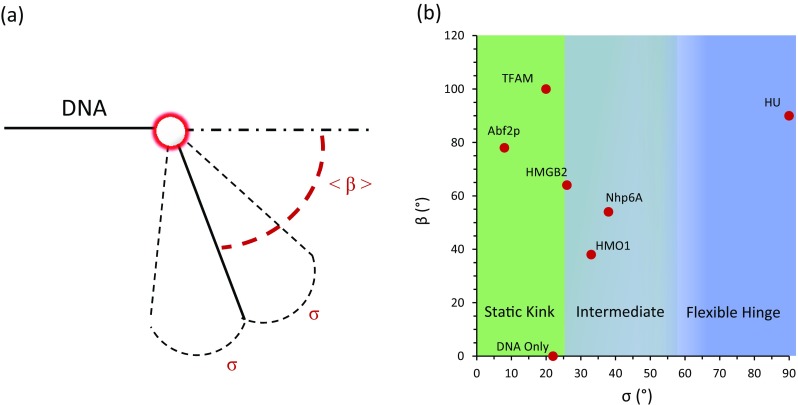
Average bend angle as a function of standard deviation. **a** Model depicting average induced DNA bend angle and its associated standard deviation. **b** The bending nature of HMGB proteins can be explained by the static kink model and a model between static kink and flexible hinge, which we refer to as intermediate

In contrast to the static kink model, the flexible hinge model proposes the creation of a flexible hinge in DNA at the site of the bound protein. β′ (in purple) represents a binding event, as shown in Fig. 7d. These irregular bends also make the DNA appear more flexible. For HU proteins, the histogram of measured local protein-induced DNA bend angles shows a broad distribution of angles and standard deviation illustrating the flexible hinge model, as shown in Fig. 7e. Although these data provide an excellent example of a pure flexible hinge protein, it is worth noting that a few other studies suggest less flexibility for HU (Kundukad et al. 2013; Sagi et al. 2004). The local flexibility around the mean bend angle β is given by the standard deviation σ, as shown in Fig. 8a. The nature of these bends with the average bend angles β along its standard deviation for both single and double box HMGB proteins are summarized in Table 2 and illustrated in Fig. 8b.

**Table 2:** Comparison of the bend angle of the single box and double box HMGB proteins Nhp6A, HMGB2, HMO1, TFAM and Afb2p

DNA Only and HMGB proteins (AFM in air)	β (°)	σ (°)	Nature of the bends	Reference
DNA only	0	24		Zhang et al. 2009
Nhp6A (single box)	54.5 ± 2.6	38.0 ± 2.0	Intermediate	Zhang et al. 2012
HMGB2 (Box A, single box)	64.5 ± 2.0	26.0 ± 1.7	Static kink	Zhang et al. 2012
HMO1 (double box)	38 ± 2	33 ± 3	Intermediate	Murugesapillai et al. 2014
TFAM (double box)	100	20	Static kink	Kaufman et al. 2007
Abf2p (double box)	78	8	Static kink	Friddle et al. 2004

The results in Table 2 suggest that HMGB proteins can generally be described either by a static kink model or as an intermediate between the static kink and flexible hinge models. One possible exception is that of TFAM, as Farge et al. (2012) concluded, based on the force dependence of protein binding, that TFAM acts as a flexible hinge. However, this is in disagreement with the results of Kaufman et al. (2007). In any case, the bulk of the results on the mechanism of DNA bending by HMGB proteins are inconsistent with the flexible hinge model initially invoked to explain slow dissociation of HMGB proteins from DNA in optical tweezers experiments (McCauley et al. 2005). Thus, a perceived discrepancy between AFM studies and optical tweezers experiments arose. Understanding and resolving this discrepancy required direct measurements of HMGB–DNA binding kinetics, which have been obtained using magnetic tweezers and fluorescence measurements. Such measurements will be discussed in the next section.

### Magnetic tweezers and fluorescence measurements reveal HMGB-DNA binding kinetics

**Fig. 9:**
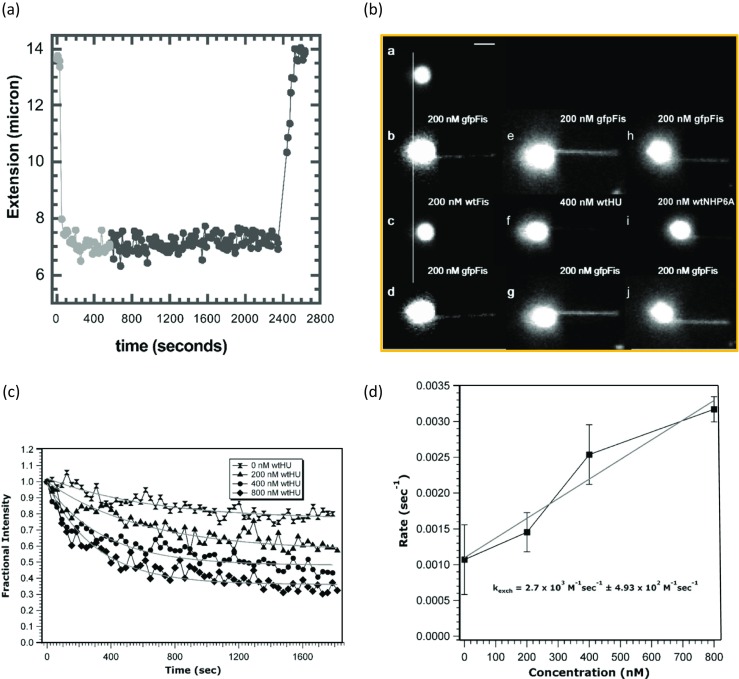
Protein-DNA off-rate in single molecule experiments is bimolecular. **a** The DNA is compacted in the presence of Nhp6A, the extension is reduced from 13.5 to 7 μm. No return of the extension was observed when buffer solution was flowed at ~600 s (transition from light to dark symbols on the graph), but after flowing competitor DNA fragments at ~2400 s, the compacted DNA recovered its initial length. **b** Fluorescence images of Fis exchange illustrates that not all proteins have exchanged. See text for description of individual frames. **c** Fluorescent protein is exchanged for non-fluorescent wild-type HU, a decrease in fluorescence intensity is observed. **d** Exchange rate obtained from each fit in (c) is proportional to concentration of wild-type HU in solution (Adapted from Skoko et al. 2004 and Graham et al. 2011, with permission)

Using magnetic tweezers to characterize HMGB protein binding to DNA, an initially perplexing result was obtained (Skoko et al. 2004). It was reported that at 0.5 pN stretching force in the presence of Nhp6A, the length of the DNA decreased from 15 to 7 μm, as shown in Fig. 9a. After ∼ 10 min, free protein was washed from the experimental chamber as previously described for optical tweezers experiments. Surprisingly, protein dissociation from DNA was not observed, and the DNA remained compacted at 7 μm. Only after free competitor DNA molecules were introduced did Nhp6A dissociate from the tethered DNA and DNA compaction was relieved, as shown in Fig. 9a. Thus, the off-rate of proteins appeared to depend on the concentration of nearby molecules (Hadizadeh et al. 2016).

Recently, it was calculated that the Nhp6A protein dissociation rate from DNA in the absence of protein in solution is extremely low, ∼10^−3^ s^−1^, such that the average protein dissociates every $\sim$16 min (McCauley et al. 2013; Skoko et al. 2004). In addition, magnetic tweezers experiments have been combined with detection of fluorescent proteins, including Nhp6A, bound to single dsDNA molecules, as shown in Fig. 9b, to probe the dissociation and exchange of DNA-bound proteins (Graham et al. 2011). In this experiment, the authors start with a DNA molecule tethered to a bead (Fig. 9b, panel a). They then introduced 200 nM gfpFis fluorescent protein (a prokaryotic architectural protein) to the chamber containing the tethered single DNA molecule, washed with 1 mL buffer, and imaged (Fig. 9b, panel b). Notably, the fluorescent protein did not dissociate from the DNA during washing. However, when unlabeled wtFis was then incubated for 3 min and again washed with 1 mL buffer and imaged, the fluorescent protein dissociated from the DNA (Fig. 9b, panel c), presumably due to exchange of unlabeled wtFis for labeled gfpFis. When gfpFis was again introduced and imaged, fluorescent protein was again observed, presumably after labeled gfpFis exchanged for unlabeled protein. The same procedure was repeated, but wtHU was observed to exchange for gfpFis (Fig. 9b, panels e–g). Finally, wtNhp6A was also exchanged readily with gfpFis (Fig. 9b, panels h–j).

It can be seen that DNA is coated by gfpFis even after buffer wash in each case, illustrating the very slow protein dissociation rate from DNA in the absence of protein in solution. Flowing wtFis (or other unlabeled proteins, such as bacterial HU and yeast Nhp6A) removed gfpFis from the DNA and from the bead (due to DNA coils on the bead). In addition, in each case, gfpFis was subsequently able to exchange with the unlabeled proteins, as observed in the bottom panels of Fig. 9b. Quantitative analysis revealed that the gfpFis dissociation rate was faster when the concentration of exchanging wtHU was increased, as shown in Fig. 9c. These experiments demonstrated a surprising linear dependence of the protein dissociation rate on free protein concentration (Fig. 9d), contradicting the standard bimolecular reaction scheme (Graham et al. 2011). The implications of these observations for HMGB protein behavior are discussed below. It is also interesting to note that it has been shown for the case of Nhp6A that there is a linear dependence of dissociation rate on the concentration of DNA, and a small dependence on the length of the DNA molecules (Aragay et al. 1988; Fried and Crothers 1984; Giuntoli et al. 2015; Menetski and Kowalczykowski 1987; Ryan and Crothers 1984; Schneider and Wetmur 1982; von Hippel and Berg 1989).

The binding and bending of HMGB protein has also been studied using single-molecule fluorescence resonance energy transfer (FRET) experiments. In those experiments, fluorescent donor and acceptor molecules were used to investigate the nature of the bends induced by Nhp6A upon DNA binding (Coats et al. 2013). Within the time resolution of the experiment, a single high FRET state was observed upon Nhp6A binding, consistent with the static kink model. The HMGB1 protein together with RAG1/2c was shown to strongly bend RSS DNA upon binding as part of V(D)J recombination (Ciubotaru et al. 2013). In addition, tethered particle motion experiments have been used to study the role of HMGB1 protein in enhancing RAG1/2c-RSS induced bending (Lovely et al. 2015).

### DNA looping proteins

**Fig. 10:**
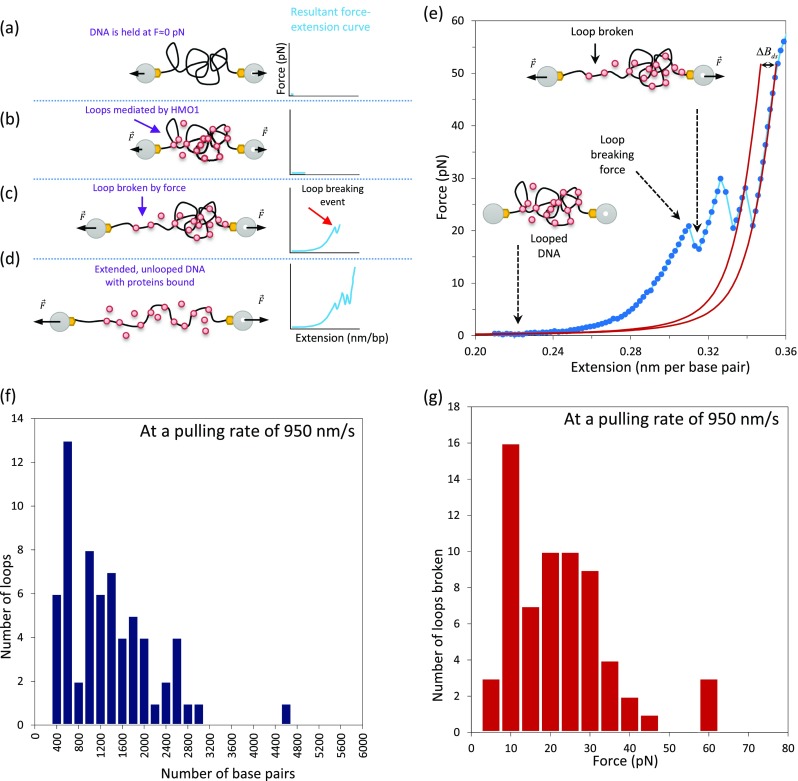
Protein–DNA loop formation as a mechanism for DNA compaction. **a**–**d** Schematic illustrating the formation and breaking of loops. When the DNA is held at low forces, HMO1 proteins are able to mediate and stabilize loops and the force–extension curve is relatively flat (**b**). Here, the *blue line* represents the force–extension curve. In contrast, when the DNA is extended further, the force–extension curve shows jumping events, revealing the breaking of loops mediated by HMO1 (**c**). As the DNA is further extended, unlooped DNA with proteins bound is stretched (**d**). **e** Force–extension curves for phage λ DNA in the presence of 0.3 nM HMO1. Each jump illustrates a loop-breaking event. Fitting is to the WLC model (*solid red lines*). Loop size is estimated by measuring the contour length change over the force jump. **f** Loop sizes. The most probable loop size is between 400 and 600 bp. **g** Loop breaking forces. The most probable loop breaking force is between 10 and 15 pN at this pulling rate of 950 nm/s. (Adapted from Murugesapillai et al. 2014)

Increased DNA flexibility in the presence of HMGB proteins enhances the probability for the DNA to cross itself, increasing the potential for DNA loop formation if proteins are present that can stabilize crossing nodes. In optical tweezers experiments, the DNA can be maintained at a very low force (F ≈ 0 pN), allowing the DNA to cross itself as shown in Fig. 10a. The presence of HMO1 proteins stabilizes these crossing nodes, as shown in Fig. 10b. To disrupt these loops, a force of few pN is applied and each jump in the experimental force–extension curve represents a loop breaking event (in blue) as illustrated in Fig. 10c. As pulling continues, the loops are broken progressively, shown in Fig. 10d. From these loop breaking events, it was possible to estimate both the DNA loop size, *ΔB*_ds_, (by fitting to the WLC model) and as well as the loop breaking force (Murugesapillai et al. 2014), as shown in Fig. 10e. The most probable loop size was found to be between 400 and 600 base pairs, shown in Fig. 10f, and the most probable breaking force was between 10 and 15 pN, although this is dependent on the pulling rate, illustrated in Fig. 10g. Interestingly, in the optical tweezers experiments it is observed that pulling at a slow rate (100 nm/s) resulted in fewer loops, suggesting that loops must form and break spontaneously. Loops were found to be stable on short time scales, reflected by the higher required force to break loops at higher pulling rates. Motors such as RNA polymerase move at ∼4 nm per second (Galburt et al. 2007) and would not be retarded by transient HMO1-mediated loops because the loops should spontaneously break on the timescale of motor movement (Murugesapillai et al. 2014).

**Fig. 11:**
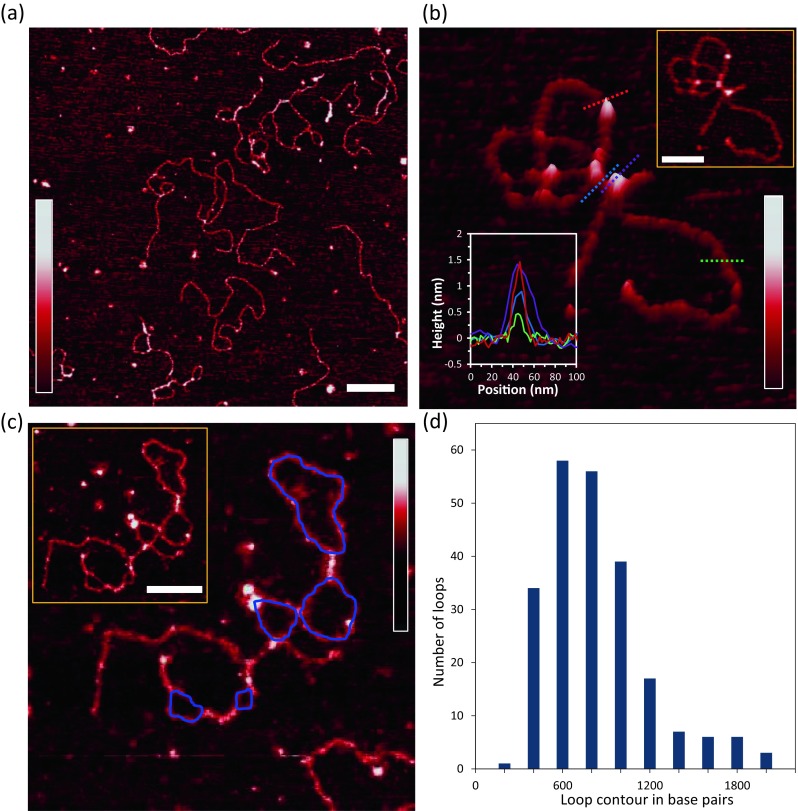
HMO1 bridges, loops and compacts DNA. **a** Two-dimensional representation of bridges and loops mediated by 3 nM HMO1 in the presence of 0.11 nM pBR322 DNA (*scale bar* 200 nm). **b** Three-dimensional representation of a looped single DNA molecule; the cross-sections of DNA only, DNA with protein-bound, protein bridging two DNA double helices, and two DNA double helices held close to each other by protein on its ends are shown in *green*, *red*, *purple* and *blue*, respectively. The *top right inset* displays a two-dimensional representation of locally probed HMO1–DNA complexes (*scale bar* 100 nm). Graphs of the heights (*bottom-left inset*) are shown for each cross-section on the image (**c**) Two-dimensional representation of HMO1 looping (protein-bound at the intersection of a loop) and bridging (protein-bound holding two strands close together) a DNA molecule. Traced loops are shown in *blue*. *Inset* Original AFM image without traces (*scale bar* 100 nm). **d** DNA loop sizes mediated by HMO1. The *color bar* *in each panel* represents the sample height ranging from 0.0 to 2.0 nm. (Adapted from Murugesapillai et al. 2014)

AFM studies also show DNA loops stabilized by HMO1, as illustrated in Fig. 11a–c. Figure 11d shows a histogram of loop sizes mediated by HMO1 in AFM experiments. The most probable loop is found between the range of 400–600 bp, similar to that observed with optical tweezers, showing that HMO1 binds to crossover nodes and stabilizes loop structures (Crampton et al. 2007; Murugesapillai et al. 2014; Neaves et al. 2009). Recent studies confirm that HMGB proteins such as TFAM and HMO1 are also able to mediate and stabilize DNA loops, a proposed mechanism for DNA compaction (Kukat et al. 2015; Murugesapillai et al. 2014). In contrast, an earlier tethered particle motion study suggested that loop formation is not mediated by TFAM (Farge et al. 2012). Further experiments are needed to resolve these discrepancies.

### Constant force measurements reveal kinetics of DNA compaction by HMGB proteins

**Fig. 12:**
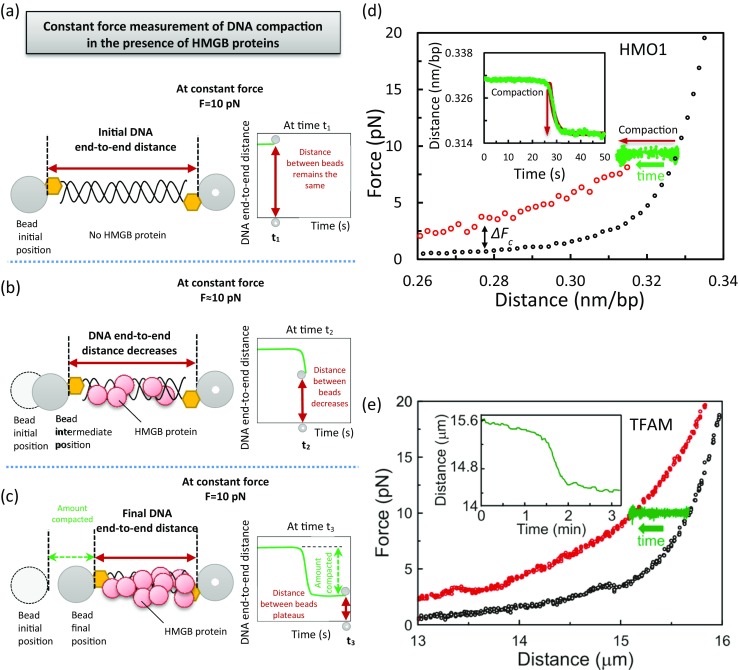
Constant force measurement. **a** A single DNA molecule is kept at a constant force of 10 pN. In the absence of proteins, the distance between beads (*red arrow*) does not change in time. **b** While keeping the force constant at 10 pN and in the presence of proteins (*red circles*), the distance between the beads decreases (*red arrow*) at a later time t_2_. **c** As the exposure time of proteins to DNA increases, the DNA molecule is further compacted and the distance between beads has further decreased and has reached a constant value (*red arrow*). The difference between the initial position and the final position of the bead indicates the total amount compacted (*green arrow*). **d** When DNA is exposed to 10 nM HMO1, constant force measurements at 10 pN and 100 nM NaCl indicate that HMO1 compacts DNA (*red arrow* pointing to the *left*). The compaction force is ∆F_c_ = 1.7 ± 0.3 pN and the rate constant for compaction, *k*, is 0.64 ± 0.10 s^−1^ (τ = 1.6 ± 0.2 s), obtained by fitting the change in extension as a function of time (*inset*, *green curve*) to a single exponential (*red line*). (Adapted from Murugesapillai et al. 2014). **e** Similarly, when DNA is exposed to 50 nM double box HMGB protein TFAM at 150 mM NaCl, the rate constant for compaction, *k*, is (3.0 ± 1.0) × 10^−2^ s^−1^ (From Farge et al. 2012, with permission)

To study the compaction of DNA by HMGB proteins, constant force optical tweezers experiments were performed. The DNA is held in a flow cell using a force feedback method to achieve a constant force of 10 pN and the DNA end-to-end distance is recorded, as shown in Fig. 12a. When HMGB proteins are introduced into the flow cell a decrease in DNA end-to-end distance is observed. This corresponds to a drop in the DNA end-to-end distance-time curve, as shown in Fig. 12b. A longer wait time resulted in the compaction of the DNA, which is indicated by the observed plateau in the DNA end-to-end distance-time curve. The change in the position of the bead illustrates the extent of DNA compaction by HMGB proteins, as shown in Fig. 12c. The experimental curves in Fig. 12d, e illustrate this method. In black is shown the extension curve of DNA in the absence of HMGB proteins. The DNA molecule is then held at 10 pN and exposed to 10 nM HMO1 in Fig. 12d (Murugesapillai et al. 2014) and 50 nM TFAM in Fig. 12e (Farge et al. 2012). In both cases, the distance between the beads was recorded over time (in green), shown in the inset in Fig. 12d, e.

**Fig. 13:**
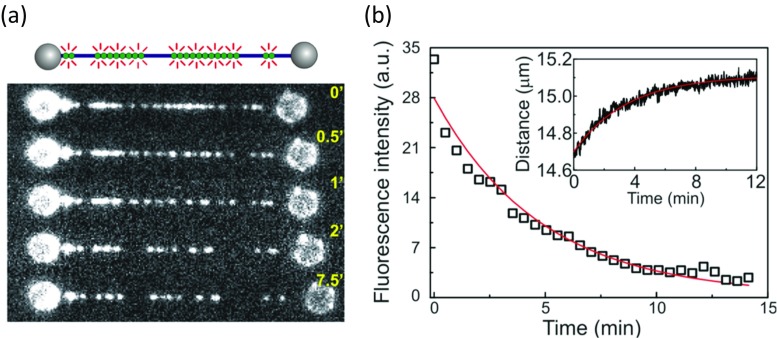
Study of 50 nM TFAM binding and unbinding events on DNA held at 10 pN. **a** The frames illustrate the unbinding events of TFAM from DNA. **b** Fluorescence intensity as a function of time for a molecule held at a constant force and covered with TFAM. The *red line* represents a fit to obtain the dissociation time. The distance between beads increases as a function of time (*inset*). (From Farge et al. 2012, with permission)

The measured extent of DNA compaction can be used to determine the compaction rate for HMO1 and TFAM. Fitting the change in the DNA end-to-end distance at constant force to a single exponential time dependence, the compaction rate constants for 10 nM HMO1 and 50 nM TFAM under these conditions were found to be *k* = 0.64 ± 0.10 s^−1^ (τ = 1.6 ± 0.2 s) and *k* = 0.074 s^−1^ (τ = 15 s), respectively (shown in red in the inset of Fig. 12d; Farge et al. 2012; Murugesapillai et al. 2014). Furthermore, dual trap optical tweezers have been combined with fluorescent TFAM proteins to investigate binding *and unbinding* events (Farge et al. 2012), as shown in Fig. 13a. In this experiment, a single molecule of DNA is kept at a constant force and exposed to protein in solution. Then the DNA is moved to a protein-free solution and frames at different time intervals are analyzed. A longer wait time resulted in an increase in unbinding events. The decay of fluorescence intensity in time allows the measurement of the dissociation rate, (3.2 ± 0.6) × 10^−3^ s^−1^ (τ = 315 ± 50 s), shown in Fig. 13b. The observed very slow dissociation rate is consistent with measurements on Nhp6A with magnetic tweezers, discussed above. The unbinding of protein has resulted in an increase in the DNA effective contour length, as shown in Fig. 13a and in the inset of Fig. 13b. To understand the very slow dissociation rates measured in these experiments, we can make use of the ability of HMGB proteins to torsionally constrain DNA. This allows us to probe the difference between microscopic and macroscopic protein dissociation rates, described in the next section.

### Torsionally constrained DNA introduced by protein binding

**Fig. 14:**
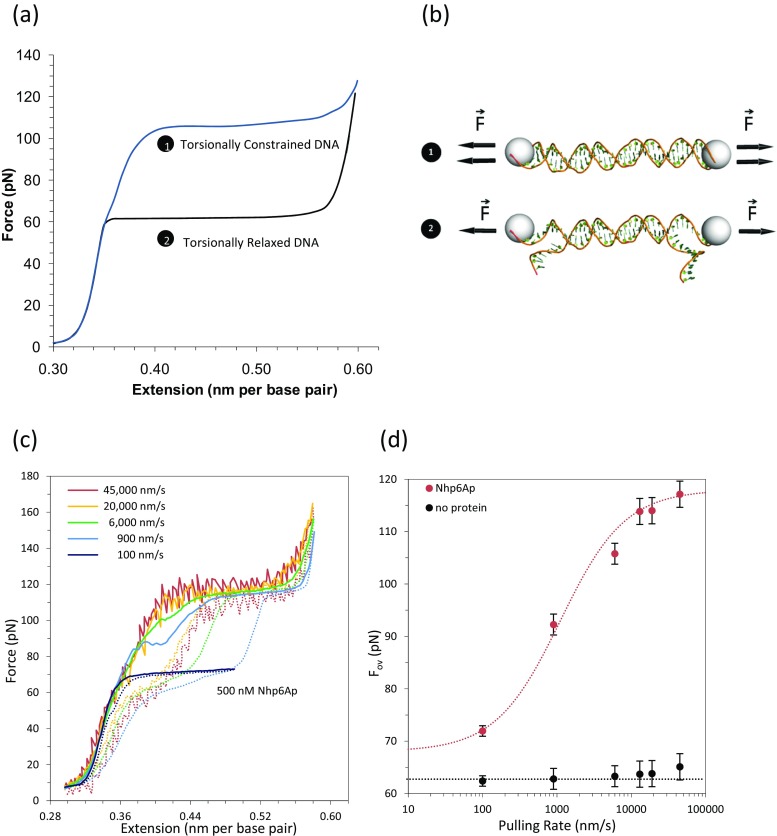
DNA torsionally constrained overstretching transition and the kinetic analysis of Nhp6A protein binding to DNA. **a** Torsionally constrained DNA is characterized by an overstretching force at 110 pN whereas torsionally relaxed DNA experiences the overstretching force at 65 pN. At high pulling rates, DNA in the presence of HMGB proteins displays an overstretching transition at forces comparable to those expected for torsionally constrained DNA. This result is interpreted as evidence that bound HMGB proteins block DNA unwinding. At low pulling rate the increase in overstretching transition due to protein binding occurs at ∼75 pN. **b** Schematic of torsionally constrained DNA (*1* unable to rotate), and torsionally unconstrained DNA (*2* able to rotate and unwind). **c** In the presence of HMGB proteins, DNA becomes torsionally constrained at pulling rates higher than the protein dissociation rate. **d** DNA overstretching force in the absence (*black*) or presence (*red*) of Nhp6A versus pulling rate, fitted to Eq. (8) to estimate the dissociation rate, *k*_*off*_. (Adapted from McCauley et al. 2013)

When single DNA molecules are tethered to beads such that only one strand is attached, the two DNA strands are free to rotate relative to one another (unwind) under a stretching force. Such DNA is therefore torsionally unconstrained. However, if both termini of each DNA strand are attached, the termini are unable to rotate, resulting in torsionally constrained DNA. Torsionally constrained DNA displays an overstretching force of 110 pN, whereas the overstretching force for torsionally unconstrained DNA is 65 pN, as illustrated in Fig. 14a, b for comparison (Leger et al. 1999; van Mameren et al. 2009; Williams et al. 2009). A detailed characterization of the biophysical properties of stretched, torsionally constrained DNA has been recently published (King et al. 2016). Here, we will use the difference in force between overstretched torsionally constrained DNA and torsionally unconstrained DNA to characterize HMGB protein–DNA interactions.

At low pulling rates in the presence of HMGB protein, the overstretching transition force of torsionally unconstrained DNA increases from 65 to ∼75 pN. It is interesting to note that at high pulling rates HMGB proteins increase the overstretching transition to forces comparable to those observed for torsionally constrained DNA, as shown in Fig. 14c. Thus, high pulling rates for DNA–HMGB protein complexes reveal the appearance of a torsionally-constrained DNA form that resists unwinding. This torsional constraint occurs on a short timescale and is not observed during slow pulling (McCauley et al. 2013).

### Measurement of microscopic protein dissociation rates

By quantifying the pulling rates at which DNA appears torsionally constrained by HMGB proteins, a *microscopic* dissociation rate was determined with optical tweezers. Rapid microscopic dissociation events had previously been undetectable because re-association was practically simultaneous. Single molecule experiments had detected apparent *macroscopic* HMGB protein dissociation rates, hundreds of times slower than would be predicted from observed equilibrium dissociation constants and binding on-rates. To quantify microscopic dissociation kinetics, the critical pulling rate, *v*_*HMGB*_, is defined as the pulling rate at which the protein naturally releases its torsional constraint during the time that the DNA is overstretched.

8 $$ {F}_{ov}(v)={F}_{ov}^L+\frac{v}{v+{v}_{HMGB}}\left({F}_{ov}^T-{F}_{ov}^L\right) $$

9 $$ {v}_{HMGB}={k}_{off, micro}N\varDelta x $$

10 $$ {k}_{off,micro}={v}_{HMGB}/N\varDelta x $$

where *F*_*ov*_^*L*^ is the protein-saturated equilibrium value observed at low pulling rates, and ${F}_{ov}^T$ is the torsionally constrained overstretching force, which is obtained in the limit of high pulling rate. *N* is the total number of base pairs and ∆*x = x*_*ss*_ − *x*_*ds*_ *=* 0.18 nm/bp is the difference in contour length between ssDNA and dsDNA. *k*_*off,micro*_ is obtained from Eq. (10) using the value for *v*_*HMGB*_ from fits of the data in Fig. 14d to Eq. (8). The measured value of *k*_*off,micro*_ is $\sim$0.1 s^−1^ for Nhp6A. This is at least two orders of magnitude faster than the macroscopic off-rate measured in prior single molecule experiments (Graham et al. 2011; Skoko et al. 2004).

Furthermore, under equilibrium conditions, the equilibrium dissociation constant can be calculated.

11 $$ DNA+ HMGB\underset{k_{off}}{\overset{k_{on}}{\rightleftharpoons }}DNA\cdot HMGB $$

12 $$ {K}_D=\frac{\left[DNA\right]\left[ HMGB\right]}{\left[DNA\cdot HMGB\right]}=\frac{k_{off}}{k_a}, $$

where the association rate constant *k*_*a*_ = *k*_*on*_/*c* has units of M^−1^s^−1^.

If it is assumed that the microscopic off-rate corresponds to the equilibrium off-rate measured in a bimolecular reaction, the dissociation rate constant for this binding reaction can also be calculated according to Eq. (12). The obtained values are consistent with those expected for a typical bimolecular reaction, as discussed below.

Here, *k*_*off,micro*_ is determined from torsionally constrained DNA stretching. The association rate *k*_a_ according to this calculation depends linearly on concentration and yields $\sim$10^6^ M^−1^s^−1^ (McCauley et al. 2013). In magnetic tweezers experiments, rapid binding has been observed near *K*_*D*_ on a time scale of τ_*on*_ $\sim$1 s (that is the time it takes for the protein to bind to a single DNA molecule when the proteins are flowed into the chamber with the DNA) (Skoko et al. 2004). Thus, from the measured association rate and the experimental value of the equilibrium dissociation constant, *K*_*D*_, the association rate constant *k*_a_ can also be estimated as

13 $$ {k}_a=\frac{1}{\tau_{on}{K}_D}, $$

yielding *k*_*a*_ $\sim$10^6^ M^−1^s^−1^. This result supports the deduction that the microscopic off-rate detected in this experiment is the rate relevant for bulk biochemical experiments (McCauley et al. 2013).

### Interpretation of kinetics results

**Fig. 15:**
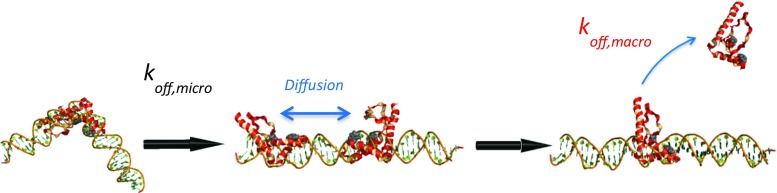
Schematic interpretation of fast microscopic dissociation versus slow macroscopic dissociation. While remaining electrostatically bound, HMG proteins locally dissociate from bent DNA at a rate described by *k*_*off,micro*_ and then diffuse along DNA before rebinding (not shown) or fully escaping from DNA at a rate described by *k*_*off,macro*_

To explain these results, it is suggested that HMGB proteins partially dissociate from local DNA binding sites and remain electrostatically bound, perhaps sliding along the DNA molecule (Fig. 15), as previously proposed (Graham et al. 2011). This model predicts that HMGB proteins will remain in contact with DNA through favorable electrostatic interactions but not be locally tightly bound. Thus, strong short-range interactions are responsible for DNA bending, and these interactions are released at a rate of ${k}_{off,micro}$, while weak long-range electrostatic interactions, which are released at a rate of ${k}_{off,macro}$, promote diffusion along DNA through loose binding (Fig. 15). The ratio of the apparent macroscopic and microscopic off-rates is ∼1000, suggesting that HMGB proteins undergo incomplete (microscopic) dissociation with local diffusion about 1000 times for each complete (macroscopic) dissociation event. Both microscopic and macroscopic rates are needed to explain the AFM, optical and magnetic tweezers results summarized here. Moreover, microscopic kinetics are revealed in the presence of free protein that acts in a competitive manner to facilitate dissociation from the DNA of protein that is loosely (macroscopically) bound. In contrast, microscopic dissociation kinetics are obscured in the absence of free protein, resulting in the appearance of anomalously slow dissociation kinetics (Graham et al. 2011; Zimmerman and Maher 2008).

## Conclusions

We have described here how equilibrium HMGB protein–DNA interactions and binding kinetics have been characterized in detail using optical tweezers, magnetic tweezers, AFM, and fluorescence imaging. These experiments have allowed quantitative insight into enhancement of DNA flexibility by different sequence-nonspecific eukaryotic HMGB proteins and functionally related prokaryotic proteins. Apparent DNA flexibility is increased through a combination of two effects. Local hinge-like flexibility can be induced in DNA at the HMGB protein binding site. In addition, stochastic protein-induced DNA bending, unbending upon protein dissociation, and re-bending upon protein re-association at new sites results in an overall average increase in sampled DNA conformations. While all HMGB proteins increase DNA flexibility, single box proteins do so more efficiently for each bound protein, typically with a greater induced DNA bending angle due to lack of co-directionality in the two bends induced by double box proteins. However, at a given protein concentration, double box proteins often have larger effects on DNA flexibility due to their higher binding affinities (Table 1). In addition to DNA flexibility enhancement, the yeast double box protein HMO1 also mediates DNA looping, which may play a role in maintaining DNA compaction in nucleosome-free chromatin regions (Murugesapillai et al. 2014).

Novel rapid pulling experiments using optical tweezers have also revealed that HMGB proteins torsionally constrain DNA on a short time scale. This has allowed sensitive measurement of a microscopic protein dissociation rate, helping to clarify protein concentration-dependent dissociation rates observed in single molecule fluorescence imaging experiments (Graham et al. 2011). Microscopic dissociation rates of 0.15 ± 0.01 and 0.88 ± 0.09 s^−1^ were measured for Nh6pA and for HMGB2 Box A, respectively. The detection of a distinct and rapid microscopic dissociation rate suggests that HMGB proteins can dissociate from a particular binding site, while remaining territorially bound to the DNA molecule. These findings emphasize that the apparent flexibility of naked DNA is increased by HMGB proteins through a constantly reorganizing ensemble of transiently-kinked HMGB–DNA complexes. These results also emphasize the importance of both strong short-range interactions (responsible for DNA binding and bending) and weak long-range electrostatic interactions (responsible for protein diffusion along DNA after partial dissociation). It is likely that these properties will characterize other non-sequence specific DNA binding proteins (Giuntoli et al. 2015; McCauley et al. 2013).

## References

[CR1] Albert B, Colleran C, Leger-Silvestre I, Berger AB, Dez C, Normand C, Perez-Fernandez J, McStay B, Gadal O (2013). Structure-function analysis of Hmo1 unveils an ancestral organization of HMG-Box factors involved in ribosomal DNA transcription from yeast to human. Nucleic Acids Res.

[CR2] Albert B, Perez-Fernandez J, Leger-Silvestre I, Gadal O (2012). Regulation of ribosomal RNA production by RNA polymerase I: does elongation come first?. Genet Res Int.

[CR3] Allain FH, Yen YM, Masse JE, Schultze P, Dieckmann T, Johnson RC, Feigon J (1999). Solution structure of the HMG protein NHP6A and its interaction with DNA reveals the structural determinants for non-sequence-specific binding. EMBO J.

[CR4] Almaqwashi AA, Paramanathan T, Rouzina I, Williams MC (2016). Mechanisms of small molecule-DNA interactions probed by single-molecule force spectroscopy. Nucleic Acids Res.

[CR5] Aragay AM, Diaz P, Daban JR (1988). Association of Nucleosome Core Particle DNA with Different Histone Oligomers - Transfer of Histones between DNA-(H2a, H2b) and DNA-(H3, H4) Complexes. J Mol Biol.

[CR6] Ashkin A, Schutze K, Dziedzic JM, Euteneuer U, Schliwa M (1990). Force generation of organelle transport measured in vivo by an infrared laser trap. Nature.

[CR7] Bauerle KT, Kamau E, Grove A (2006). Interactions between N- and C-terminal domains of the Saccharomyces cerevisiae high-mobility group protein HMO1 are required for DNA bending. Biochemistry.

[CR8] Baumann CG, Smith SB, Bloomfield VA, Bustamante C (1997). Ionic effects on the elasticity of single DNA molecules. Proc Natl Acad Sci U S A.

[CR9] Berger AB, Decourty L, Badis G, Nehrbass U, Jacquier A, Gadal O (2007). Hmo1 is required for TOR-dependent regulation of ribosomal protein gene transcription. Mol Cell Biol.

[CR10] Bianchi ME (2009). HMGB1 loves company. J Leukoc Biol.

[CR11] Bianchi ME, Agresti A (2005). HMG proteins: dynamic players in gene regulation and differentiation. Curr Opin Genet Dev.

[CR12] Bianco P, Bongini L, Melli L, Dolfi M, Lombardi V (2011). PicoNewton-millisecond force steps reveal the transition kinetics and mechanism of the double-stranded DNA elongation. Biophys J.

[CR13] Biebricher AS, Heller I, Roijmans RFH, Hoekstra TP, Peterman EJG, Wuite GJL (2015) The impact of DNA intercalators on DNA and DNA-processing enzymes elucidated through force-dependent binding kinetics. Nat Commun 6 doi: 10.1038/ncomms8304 10.1038/ncomms8304 PMC4557362 26084388

[CR14] Bogenhagen DF, Rousseau D, Burke S (2008). The layered structure of human mitochondrial DNA nucleoids. J Biol Chem.

[CR15] Bogenhagen DF, Wang Y, Shen EL, Kobayashi R (2003). Protein components of mitochondrial DNA nucleoids in higher eukaryotes. Mol Cell Proteomics.

[CR16] Bongini L, Lombardi V, Bianco P (2014). The transition mechanism of DNA overstretching: a microscopic view using molecular dynamics. J R Soc Interface.

[CR17] Bongini L, Melli L, Lombardi V, Bianco P (2014). Transient kinetics measured with force steps discriminate between double-stranded DNA elongation and melting and define the reaction energetics. Nucleic Acids Res.

[CR18] Bosaeus N, El-Sagheer AH, Brown T, Akerman B, Norden B (2014). Force-induced melting of DNA--evidence for peeling and internal melting from force spectra on short synthetic duplex sequences. Nucleic Acids Res.

[CR19] Bosaeus N, El-Sagheer AH, Brown T, Smith SB, Akerman B, Bustamante C, Norden B (2012). Tension induces a base-paired overstretched DNA conformation. Proc Natl Acad Sci U S A.

[CR20] Brewer LR, Friddle R, Noy A, Baldwin E, Martin SS, Corzett M, Balhorn R, Baskin RJ (2003). Packaging of single DNA molecules by the yeast mitochondrial protein Abf2p. Biophys J.

[CR21] Bustamante C, Bryant Z, Smith SB (2003). Ten years of tension: single-molecule DNA mechanics. Nature.

[CR22] Chaurasiya KR, Paramanathan T, McCauley MJ, Williams MC (2010). Biophysical characterization of DNA binding from single molecule force measurements. Phys Life Rev.

[CR23] Chen H, Fu H, Zhu X, Cong P, Nakamura F, Yan J (2011). Improved high-force magnetic tweezers for stretching and refolding of proteins and short DNA. Biophys J.

[CR24] Cho JH, Lee YK, Chae CB (2001). The modulation of the biological activities of mitochondrial histone Abf2p by yeast PKA and its possible role in the regulation of mitochondrial DNA content during glucose repression. Biochim Biophys Acta.

[CR25] Churchill ME, Changela A, Dow LK, Krieg AJ (1999). Interactions of high mobility group box proteins with DNA and chromatin. Methods Enzymol.

[CR26] Ciubotaru M, Trexler AJ, Spiridon LN, Surleac MD, Rhoades E, Petrescu AJ, Schatz DG (2013). RAG and HMGB1 create a large bend in the 23RSS in the V(D)J recombination synaptic complexes. Nucleic Acids Res.

[CR27] Cluzel P, Lebrun A, Heller C, Lavery R, Viovy JL, Chatenay D, Caron F (1996). DNA: an extensible molecule. Science.

[CR28] Coats JE, Lin Y, Rueter E, Maher LJ, Rasnik I (2013). Single-molecule FRET analysis of DNA binding and bending by yeast HMGB protein Nhp6A. Nucleic Acids Res.

[CR29] Crampton N, Roes S, Dryden DT, Rao DN, Edwardson JM, Henderson RM (2007). DNA looping and translocation provide an optimal cleavage mechanism for the type III restriction enzymes. EMBO J.

[CR30] Crothers DM (1993). Architectural elements in nucleoprotein complexes. Curr Biol.

[CR31] Cruceanu M, Urbaneja MA, Hixson CV, Johnson DG, Datta SA, Fivash MJ, Stephen AG, Fisher RJ, Gorelick RJ, Casas-Finet JR, Rein A, Rouzina I, Williams MC (2006). Nucleic acid binding and chaperone properties of HIV-1 Gag and nucleocapsid proteins. Nucleic Acids Res.

[CR32] Dame RT, van Mameren J, Luijsterburg MS, Mysiak ME, Janicijevic A, Pazdzior G, van der Vliet PC, Wyman C, Wuite GJ (2005). Analysis of scanning force microscopy images of protein-induced DNA bending using simulations. Nucleic Acids Res.

[CR33] De Vlaminck I, Dekker C (2012). Recent advances in magnetic tweezers. Annu Rev Biophys.

[CR34] Diffley JF, Stillman B (1992). DNA binding properties of an HMG1-related protein from yeast mitochondria. J Biol Chem.

[CR35] Dragan AI, Klass J, Read C, Churchill ME, Crane-Robinson C, Privalov PL (2003). DNA binding of a non-sequence-specific HMG-D protein is entropy driven with a substantial non-electrostatic contribution. J Mol Biol.

[CR36] Dragan AI, Read CM, Makeyeva EN, Milgotina EI, Churchill ME, Crane-Robinson C, Privalov PL (2004). DNA binding and bending by HMG boxes: energetic determinants of specificity. J Mol Biol.

[CR37] Farge G, Laurens N, Broekmans OD, van den Wildenberg SM, Dekker LC, Gaspari M, Gustafsson CM, Peterman EJ, Falkenberg M, Wuite GJ (2012). Protein sliding and DNA denaturation are essential for DNA organization by human mitochondrial transcription factor. A Nat Commun.

[CR38] Friddle RW, Klare JE, Martin SS, Corzett M, Balhorn R, Baldwin EP, Baskin RJ, Noy A (2004). Mechanism of DNA compaction by yeast mitochondrial protein Abf2p. Biophys J.

[CR39] Fried MG, Crothers DM (1984). Kinetics and Mechanism in the Reaction of Gene Regulatory Proteins with DNA. J Mol Biol.

[CR40] Fu H, Chen H, Marko JF, Yan J (2010). Two distinct overstretched DNA states. Nucleic Acids Res.

[CR41] Galburt EA, Grill SW, Wiedmann A, Lubkowska L, Choy J, Nogales E, Kashlev M, Bustamante C (2007). Backtracking determines the force sensitivity of RNAP II in a factor-dependent manner. Nature.

[CR42] Gerlitz G, Hock R, Ueda T, Bustin M (2009). The dynamics of HMG protein-chromatin interactions in living cells. Biochem Cell Biol.

[CR43] Giuntoli RD, Linzer NB, Banigan EJ, Sing CE, de la Cruz MO, Graham JS, Johnson RC, Marko JF (2015). DNA-Segment-Facilitated Dissociation of Fis and NHP6A from DNA Detected via Single-Molecule Mechanical Response. J Mol Biol.

[CR44] Gosse C, Croquette V (2002). Magnetic tweezers: micromanipulation and force measurement at the molecular level. Biophys J.

[CR45] Graham JS, Johnson RC, Marko JF (2011). Concentration-dependent exchange accelerates turnover of proteins bound to double-stranded DNA. Nucleic Acids Res.

[CR46] Gross P, Laurens N, Oddershede LB, Bockelmann U, Peterman EJG, Wuite GJL (2011). Quantifying how DNA stretches, melts and changes twist under tension. Nat Phys.

[CR47] Hadizadeh N, Johnson RC, Marko JF (2016). Facilitated Dissociation of a Nucleoid Protein from the Bacterial Chromosome. J Bacteriol.

[CR48] Hall DB, Wade JT, Struhl K (2006). An HMG protein, Hmo1, associates with promoters of many ribosomal protein genes and throughout the rRNA gene locus in Saccharomyces cerevisiae. J Biol Chem.

[CR49] Heller I, Hoekstra TP, King GA, Peterman EJ, Wuite GJ (2014). Optical tweezers analysis of DNA-protein complexes. Chem Rev.

[CR50] Heller I, Sitters G, Broekmans OD, Farge G, Menges C, Wende W, Hell SW, Peterman EJ, Wuite GJ (2013). STED nanoscopy combined with optical tweezers reveals protein dynamics on densely covered DNA. Nat Methods.

[CR51] Kamau E, Bauerle KT, Grove A (2004). The Saccharomyces cerevisiae high mobility group box protein HMO1 contains two functional DNA binding domains. J Biol Chem.

[CR52] Kang D, Kim SH, Hamasaki N (2007). Mitochondrial transcription factor A (TFAM): roles in maintenance of mtDNA and cellular functions. Mitochondrion.

[CR53] Kaufman BA, Durisic N, Mativetsky JM, Costantino S, Hancock MA, Grutter P, Shoubridge EA (2007). The mitochondrial transcription factor TFAM coordinates the assembly of multiple DNA molecules into nucleoid-like structures. Mol Biol Cell.

[CR54] King GA, Gross P, Bockelmann U, Modesti M, Wuite GJ, Peterman EJ (2013). Revealing the competition between peeled ssDNA, melting bubbles, and S-DNA during DNA overstretching using fluorescence microscopy. Proc Natl Acad Sci U S A.

[CR55] King GA, Peterman EJ, Wuite GJ (2016). Unravelling the structural plasticity of stretched DNA under torsional constraint. Nat Commun.

[CR56] Klass J, Murphy FV, Fouts S, Serenil M, Changela A, Siple J, Churchill ME (2003). The role of intercalating residues in chromosomal high-mobility-group protein DNA binding, bending and specificity. Nucleic Acids Res.

[CR57] Kowalczykowski SC, Paul LS, Lonberg N, Newport JW, McSwiggen JA, von Hippel PH (1986). Cooperative and noncooperative binding of protein ligands to nucleic acid lattices: experimental approaches to the determination of thermodynamic parameters. Biochemistry.

[CR58] Kukat C, Davies KM, Wurm CA, Spahr H, Bonekamp NA, Kuhl I, Joos F, Polosa PL, Park CB, Posse V, Falkenberg M, Jakobs S, Kuhlbrandt W, Larsson NG (2015). Cross-strand binding of TFAM to a single mtDNA molecule forms the mitochondrial nucleoid. Proc Natl Acad Sci U S A.

[CR59] Kundukad B, Cong P, van der Maarel JR, Doyle PS (2013). Time-dependent bending rigidity and helical twist of DNA by rearrangement of bound HU protein. Nucleic Acids Res.

[CR60] Lange SS, Mitchell DL, Vasquez KM (2008). High mobility group protein B1 enhances DNA repair and chromatin modification after DNA damage. Proc Natl Acad Sci U S A.

[CR61] Leger JF, Romano G, Sarkar A, Robert J, Bourdieu L, Chatenay D, Marko JF (1999). Structural transitions of a twisted and stretched DNA molecule. Phys Rev Lett.

[CR62] Liu Y, Prasad R, Wilson SH (2010). HMGB1: roles in base excision repair and related function. Biochim Biophys Acta.

[CR63] Lodeiro MF, Uchida A, Bestwick M, Moustafa IM, Arnold JJ, Shadel GS, Cameron CE (2012). Transcription from the second heavy-strand promoter of human mtDNA is repressed by transcription factor A in vitro. Proc Natl Acad Sci U S A.

[CR64] Lovely GA, Brewster RC, Schatz DG, Baltimore D, Phillips R (2015). Single-molecule analysis of RAG-mediated V(D)J DNA cleavage. Proc Natl Acad Sci U S A.

[CR65] Malarkey CS, Churchill ME (2012). The high mobility group box: the ultimate utility player of a cell. Trends Biochem Sci.

[CR66] Marko JF, Siggia ED (1995). Stretching DNA. Macromolecules.

[CR67] McCauley M, Hardwidge PR, Maher LJ, Williams MC (2005). Dual binding modes for an HMG domain from human HMGB2 on DNA. Biophys J.

[CR68] McCauley MJ, Rueter EM, Rouzina I, Maher LJ, Williams MC (2013). Single-molecule kinetics reveal microscopic mechanism by which High-Mobility Group B proteins alter DNA flexibility. Nucleic Acids Res.

[CR69] McCauley MJ, Shokri L, Sefcikova J, Venclovas C, Beuning PJ, Williams MC (2008). Distinct double- and single-stranded DNA binding of E. coli replicative DNA polymerase III alpha subunit. ACS Chem Biol.

[CR70] McCauley MJ, Williams MC (2009). Optical tweezers experiments resolve distinct modes of DNA-protein binding. Biopolymers.

[CR71] McCauley MJ, Williams MC (2011). Measuring DNA-Protein Binding Affinity on a Single Molecule Using Optical Tweezers. Methods Mol Biol.

[CR72] McCauley MJ, Zimmerman J, Maher LJ, Williams MC (2007). HMGB binding to DNA: single and double box motifs. J Mol Biol.

[CR73] McGhee JD (1976). Theoretical calculations of the helix-coil transition of DNA in the presence of large, cooperatively binding ligands. Biopolymers.

[CR74] McGhee JD, von Hippel PH (1974). Theoretical aspects of DNA-protein interactions: co-operative and non-co-operative binding of large ligands to a one-dimensional homogeneous lattice. J Mol Biol.

[CR75] Menetski JP, Kowalczykowski SC (1987). Transfer of recA protein from one polynucleotide to another. Kinetic evidence for a ternary intermediate during the transfer reaction. J Biol Chem.

[CR76] Merz K, Hondele M, Goetze H, Gmelch K, Stoeckl U, Griesenbeck J (2008). Actively transcribed rRNA genes in S. cerevisiae are organized in a specialized chromatin associated with the high-mobility group protein Hmo1 and are largely devoid of histone molecules. Genes Dev.

[CR77] Murphy FV, Sweet RM, Churchill ME (1999). The structure of a chromosomal high mobility group protein-DNA complex reveals sequence-neutral mechanisms important for non-sequence-specific DNA recognition. EMBO J.

[CR78] Murugesapillai D, McCauley MJ, Huo R, Nelson Holte MH, Stepanyants A, Maher LJ, Israeloff NE, Williams MC (2014). DNA bridging and looping by HMO1 provides a mechanism for stabilizing nucleosome-free chromatin. Nucleic Acids Res.

[CR79] Neaves KJ, Cooper LP, White JH, Carnally SM, Dryden DT, Edwardson JM, Henderson RM (2009). Atomic force microscopy of the EcoKI Type I DNA restriction enzyme bound to DNA shows enzyme dimerization and DNA looping. Nucleic Acids Res.

[CR80] Neuman KC, Block SM (2004). Optical trapping. Rev Sci Instrum.

[CR81] Odijk T (1995). Stiff Chains and Filaments under Tension. Macromolecules.

[CR82] Paik DH, Perkins TT (2011). Overstretching DNA at 65 pN does not require peeling from free ends or nicks. J Am Chem Soc.

[CR83] Parisi MA, Xu B, Clayton DA (1993). A human mitochondrial transcriptional activator can functionally replace a yeast mitochondrial HMG-box protein both in vivo and in vitro. Mol Cell Biol.

[CR84] Paull TT, Carey M, Johnson RC (1996). Yeast HMG proteins NHP6A/B potentiate promoter-specific transcriptional activation in vivo and assembly of preinitiation complexes in vitro. Genes Dev.

[CR85] Paull TT, Haykinson MJ, Johnson RC (1993). The nonspecific DNA-binding and -bending proteins HMG1 and HMG2 promote the assembly of complex nucleoprotein structures. Genes Dev.

[CR86] Pil PM, Chow CS, Lippard SJ (1993). High-mobility-group 1 protein mediates DNA bending as determined by ring closures. Proc Natl Acad Sci U S A.

[CR87] Podgornik R, Hansen PL, Parsegian VA (2000). Elastic moduli renormalization in self-interacting stretchable polyelectrolytes. J Chem Phys.

[CR88] Ragab A, Travers A (2003). HMG-D and histone H1 alter the local accessibility of nucleosomal DNA. Nucleic Acids Res.

[CR89] Rivetti C, Codeluppi S (2001). Accurate length determination of DNA molecules visualized by atomic force microscopy: evidence for a partial B- to A-form transition on mica. Ultramicroscopy.

[CR90] Rivetti C, Guthold M, Bustamante C (1996). Scanning force microscopy of DNA deposited onto mica: equilibration versus kinetic trapping studied by statistical polymer chain analysis. J Mol Biol.

[CR91] Ross ED, Hardwidge PR, Maher LJ (2001). HMG proteins and DNA flexibility in transcription activation. Mol Cell Biol.

[CR92] Rouzina I, Bloomfield VA (1998). DNA bending by small, mobile multivalent cations. Biophys J.

[CR93] Rubio-Cosials A, Solà M (2013) U-turn DNA bending by human mitochondrial transcription factor A. Current Opin Struct Biol 23(1):116–124 10.1016/j.sbi.2012.12.004 23333034

[CR94] Ryan DP, Crothers DM (1984). Relaxation Kinetics of DNA-Ligand Binding Including Direct Transfer. Biopolymers.

[CR95] Sagi D, Friedman N, Vorgias C, Oppenheim AB, Stavans J (2004). Modulation of DNA conformations through the formation of alternative high-order HU-DNA complexes. J Mol Biol.

[CR96] Schellman JA (1974). Flexibility of DNA. Biopolymers.

[CR97] Schneider RJ, Wetmur JG (1982). Kinetics of transfer of Escherichia coli single strand deoxyribonucleic acid binding protein between single-stranded deoxyribonucleic acid molecules. Biochemistry.

[CR98] Sebastian NT, Bystry EM, Becker NA, Maher LJ (2009). Enhancement of DNA flexibility in vitro and in vivo by HMGB box A proteins carrying box B residues. Biochemistry.

[CR99] Shokri L, McCauley MJ, Rouzina I, Williams MC (2008). DNA overstretching in the presence of glyoxal: structural evidence of force-induced DNA melting. Biophys J.

[CR100] Skoko D, Wong B, Johnson RC, Marko JF (2004). Micromechanical analysis of the binding of DNA-bending proteins HMGB1, NHP6A, and HU reveals their ability to form highly stable DNA-protein complexes. Biochemistry.

[CR101] Smith SB, Cui Y, Bustamante C (1996). Overstretching B-DNA: the elastic response of individual double-stranded and single-stranded DNA molecules. Science.

[CR102] Spelbrink JN (2010). Functional organization of mammalian mitochondrial DNA in nucleoids: history, recent developments, and future challenges. IUBMB Life.

[CR103] Stefanovsky VY, Pelletier G, Bazett-Jones DP, Crane-Robinson C, Moss T (2001). DNA looping in the RNA polymerase I enhancesome is the result of non-cooperative in-phase bending by two UBF molecules. Nucleic Acids Res.

[CR104] Storm C, Nelson PC (2003). Theory of high-force DNA stretching and overstretching. Phys Rev E.

[CR105] Štros M (2010). HMGB proteins: Interactions with DNA and chromatin. BBA-Gene Regul Mech.

[CR106] Thomas JO, Travers AA (2001). HMG1 and 2, and related ‘architectural’ DNA-binding proteins. Trends Biochem Sci.

[CR107] Travers AA (2003). Priming the nucleosome: a role for HMGB proteins?. EMBO Rep.

[CR108] van Mameren J, Gross P, Farge G, Hooijman P, Modesti M, Falkenberg M, Wuite GJ, Peterman EJ (2009). Unraveling the structure of DNA during overstretching by using multicolor, single-molecule fluorescence imaging. Proc Natl Acad Sci U S A.

[CR109] van Noort J, Verbrugge S, Goosen N, Dekker C, Dame RT (2004). Dual architectural roles of HU: formation of flexible hinges and rigid filaments. Proc Natl Acad Sci U S A.

[CR110] von Hippel PH, Berg OG (1989). Facilitated Target Location in Biological-Systems. J Biol Chem.

[CR111] Venema J, Tollervey D (1999). Ribosome synthesis in Saccharomyces cerevisiae. Annu Rev Genet.

[CR112] Vologodskii A (2015) Biophysics of DNA. Cambridge University Press, Cambridge

[CR113] Wenner JR, Williams MC, Rouzina I, Bloomfield VA (2002). Salt dependence of the elasticity and overstretching transition of single DNA molecules. Biophys J.

[CR114] Wiggins PA, van der Heijden T, Moreno-Herrero F, Spakowitz A, Phillips R, Widom J, Dekker C, Nelson PC (2006). High flexibility of DNA on short length scales probed by atomic force microscopy. Nat Nanotechnol.

[CR115] Williams MC, Rouzina I, Bloomfield VA (2002). Thermodynamics of DNA interactions from single molecule stretching experiments. Acc Chem Res.

[CR116] Williams MC, Rouzina I, McCauley MJ (2009). Peeling back the mystery of DNA overstretching. Proc Natl Acad Sci U S A.

[CR117] Williams MC, Wenner JR, Rouzina I, Bloomfield VA (2001). Effect of pH on the overstretching transition of double-stranded DNA: evidence of force-induced DNA melting. Biophys J.

[CR118] Williams MC, Wenner JR, Rouzina I, Bloomfield VA (2001). Entropy and heat capacity of DNA melting from temperature dependence of single molecule stretching. Biophys J.

[CR119] Wittner M, Hamperl S, Stockl U, Seufert W, Tschochner H, Milkereit P, Griesenbeck J (2011). Establishment and maintenance of alternative chromatin states at a multicopy gene locus. Cell.

[CR120] Xiao B, Johnson RC, Marko JF (2010) Modulation of HU-DNA interactions by salt concentration and applied force. Nucleic Acids Res 38(18):6176–6185 10.1093/nar/gkq435 PMC2952867 20497998

[CR121] Zhang J, McCauley MJ, Maher LJ, Williams MC, Israeloff NE (2009). Mechanism of DNA flexibility enhancement by HMGB proteins. Nucleic Acids Res.

[CR122] Zhang J, McCauley MJ, Maher LJ, Williams MC, Israeloff NE (2012). Basic N-Terminus of Yeast Nhp6A Regulates the Mechanism of Its DNA Flexibility Enhancement. J Mol Biol.

[CR123] Zhang X, Chen H, Le S, Rouzina I, Doyle PS, Yan J (2013). Revealing the competition between peeled ssDNA, melting bubbles, and S-DNA during DNA overstretching by single-molecule calorimetry. Proc Natl Acad Sci U S A.

[CR124] Zimmerman J, Maher LJ (2008). Transient HMGB protein interactions with B-DNA duplexes and complexes. Biochem Biophys Res Commun.

